# Antioxidant and anti-inflammatory properties of water kefir microbiota and its bioactive metabolites for health promoting bio-functional products and applications

**DOI:** 10.3934/microbiol.2024034

**Published:** 2024-09-05

**Authors:** Dimitra Papadopoulou, Vasiliki Chrysikopoulou, Aikaterini Rampaouni, Alexandros Tsoupras

**Affiliations:** Hephaestus Laboratory, School of Chemistry, Faculty of Science, Democritus University of Thrace, Kavala University Campus, 65404, Kavala, Greece

**Keywords:** water kefir, lactobacillus, acetobacter, bifidobacteria, zymomonas, fungi, yeasts, anti-inflammatory, anti-oxidant, health benefits, functional products

## Abstract

Inflammation and oxidative stress are implicated in several chronic disorders, while healthy foods and especially fermented beverages and those containing probiotics can provide anti-inflammatory and antioxidant protection against such manifestations and the associated disorders. Water kefir is such a beverage that is rich in both probiotic microbiota and anti-inflammatory bioactives, with an increasing demand as an alternative to a fermented product based on non-dairy matrix with potential health properties. Within this study, the health-promoting properties of the most representative species and strains of microorganisms present in water kefir grains, as well as the health benefits attributed to the bioactive metabolites produced by each individual strain in a series of their cultures, were thoroughly reviewed. Emphasis was given to the antioxidant, antithrombotic, and anti-inflammatory bio-functionalities of both the cultured microorganisms and the bioactive metabolites produced in each case. Moreover, an extensive presentation of the antioxidant and anti-inflammatory health benefits observed from the overall water kefir cultures and classic water kefir beverages obtained were also conducted. Finally, the use of water kefir for the production of several other bio-functional products, including fermented functional foods, supplements, nutraceuticals, nutricosmetics, cosmeceuticals, and cosmetic applications with anti-inflammatory and antioxidant health promoting potential was also thoroughly discussed. Limitations and future perspectives on the use of water kefir, its microorganisms, and their bioactive metabolites are also outlined.

## 1. Introduction

According to the latest World Health Organization report (World Health Statistics 2023: Monitoring health for the SDGs, sustainable development goals), most deaths in 2019 (about 30–35 million deaths) were caused by specific chronic diseases, such as cardiovascular disease (17.9 million deaths), cancer (9.3 million deaths), chronic respiratory disease (4.1 million deaths), and diabetes (2 million deaths) [1], especially in the developed and developing countries, where there is an increased incidence of deaths from such disorders [2],[3]. Furthermore, patients with such illnesses have their quality of life and life expectancy undermined. Consequently, both the health systems and the economies of such countries and of the organizations to which they belong (i.e., the European Union) are burdened.

Several risk factors have been found to be associated with the development of such conditions, which can be either non-modifiable (i.e., age, gender, hereditary history.) or modifiable (i.e., unhealthy diet, smoking, sedentary lifestyle, obesity, increased alcohol consumption). The latter are influenced by specific lifestyles and unhealthy habits, mainly observed in people living in developed and developing countries, and are associated with increased mortality in these societies [4],[5]. It has been found that the constant presence of such risk factors induces continuous and unresolved oxidative stress, inflammatory responses, and associated manifestations that promote chronic diseases [6]–[8].

Inflammation is a physiological process of the body that is activated in response to exogenous factors (e.g., infection) and/or injury (e.g., tissue damage) in order to counterattack the insulting agent and facilitate the restoration of the homeostasis of affected tissues. However, if left unresolved, inflammation can become chronic, causing the pathogenesis of several chronic disorders [7],[9]. Chronic inflammation can induce and/or be induced by oxidative stress in a vicious cycle that usually results in chronic diseases, including the aforementioned ones. Oxidative stress is caused by the production and action of Reactive Oxygen Species (ROS) and their reduced deactivation by the body's innate antioxidant defense mechanisms. During oxidative stress-derived complications, the cellular antioxidant system is overwhelmed by the overproduction of ROS. The role of ROS is twofold: In normal amounts, they are essential for vascular homeostasis, but their uncontrolled production leads to various complications, such as vascular damage [10],[11]. Inflammatory signaling pathways can induce oxidative stress and vice-versa, while their interaction induces thrombo-inflammatory complications that usually result in the aforementioned chronic diseases [6],[7].

The appropriate treatment for such major chronic diseases is administering specific medication for each condition. However, preventing such manifestations is also important and can be achieved through the appropriate modification of modifiable risk factors, including adopting healthy eating habits, engaging in regular physical activity, abstaining from alcohol abuse and smoking, managing stress appropriately, etc. From these, adopting a healthy diet seems to have a major impact, as healthy eating patterns, such as the Mediterranean diet, are beneficial against oxidative stress and inflammation and, by extension, against several inflammation-related chronic diseases [7],[12],[13]. Nevertheless, the demands of modern lifestyles complicate the realistic adoption of healthy eating habits, which leads to the need for finding other solutions, such as the intake of nutrients and bioactive ingredients of natural origin from various sources, through dietary supplements or other health-promoting bio-functional products (i.e., Functional Foods) [14].

This general context has helped the development and commercialization of healthy beverages that ideally contain minerals, vitamins, anti-inflammatory compounds, a high content of antioxidants, and a low sugar and alcohol content. Fermented products and the microorganisms involved in such processes are of interest [15]–[18] as they possess many bioactive metabolites, while such microflora can also benefit the gut microbiota with subsequent pleiotropic health-promoting effects. Thus, the use of fermentation and the presence of live microorganisms that can positively affect the consumer's health, such as probiotic bacteria and yeasts, could contribute to offering consumers better choices in terms of bio-functional beverages. Fermentation technology could improve product properties and attenuate chronic inflammation by increasing the microbial diversity in the host digestive system. Within this concept, many microorganisms (MO) of biotechnological and agri-food interest seem to be viable sources of biologically active ingredients for nutritional supplements, functional foods, cosmetics, or even drugs, with anti-inflammatory and antioxidant benefits [15],[19]–[22]. Several species of yeasts and generally fermenting microorganisms, have been used to enrich foods, especially fermented foods, as well as food supplements and nutraceuticals with their bioactive ingredients [20]. Furthermore, yeasts and their bioactive metabolites can be used in such applications, conferring beneficial health-promoting effects on the obtained products [20].

Kefir, a fermented beverage made from kefir grains, which contain a mixture of probiotics, has gained immense popularity due to its proposed health-promoting properties [23]. Both milk and sugar solutions can be fermented by kefir grains with various additives to produce several products based on consumer preference. Fermentation occurs via microorganisms, including lactic acid bacteria, acetic acid bacteria, and yeasts, which are naturally present in kefir grains. The health-promoting effects of kefir and its bioactive metabolites are thought to occur through immune, gastrointestinal, and metabolic regulation. More specifically, outcomes from *in vitro* and *in vivo* studies, both in animal models and from clinical trials, have shown that kefir bioactive components can reduce proinflammatory cytokine production, contribute to the cytotoxicity of the tumor cell lines, reduce tumor burden, and improve serum glycemic and lipid profiles. However, some data from clinical trials are conflicting, and the precise mechanisms by which kefir compounds promote well-being are not completely defined [24].

In this review, the current body of evidence for the anti-inflammatory, antioxidant, and antithrombotic bioactive components of microorganisms and their strains, which have been found in water kefir, as well as their health-promoting effects in several products, cases, and experimental models, are thoroughly outlined. Apart from solely reviewing each strain of these microorganisms in several products, emphasis is given to their potential application in kefir and in its water-based cultures and the bioactives produced during the fermentation processes taking place to produce bio-functional products, such as fermented products, functional foods, nutraceuticals, nutricosmetics, cosmeceuticals, and cosmetics or pharmaceuticals, with antioxidant and anti-inflammatory health-promoting properties for the prevention of chronic diseases. We summarize the outcomes derived from both *in vitro* cell culture-based studies and *in vivo* studies in animal models and clinical trials that provide insight into the health-promoting properties of water kefir, its microbiota, and the kefir-derived functional products, along with proposed mechanisms by which kefir products and their bioactives improve immune and metabolic health. The knowledge gained and the selection of informative outcomes for the bioactive content of kefir microorganisms can promote the design and development of kefir-derived functional products with anti-inflammatory and antioxidant potential when designing more targeted mechanistic studies and clinical trials.

## 2. Microorganisms of Water kefir and their bioactive metabolites with potential health promoting properties

Kefir is an ancient, handmade, acidic beverage obtained by the fermentation of liquid culture media from kefir grains using milk as the optimal medium but also sugary water solutions, the production of which has now been industrialized and commercialized [25]. Kefir grains consist of a symbiotic colony of microorganisms that is attached to a polysaccharide gel. The microflora of the kefir grains includes *lactic acid-active bacteria* (LAB), *acetate bacteria* (AAB), *Bifidobacteria*, and several other bacteria like *Zymomonas mobilis*, as well as some fungi and yeasts, all of which vary according to the sample location [26]. These microorganisms coexist in the granules, and some can be transferred to the liquid phase. In addition, they are non-pathogenic, and, in combination with the metabolites they produce, which are present in the final fermentation product, they induce a variety of health-promoting effects, such as probiotic properties [25],[27], regulating the composition of the intestinal microflora and low-grade inflammation [28], exhibiting antimicrobial properties by inhibiting the growth of various pathogenic microorganisms [29], and improving overall immune and metabolic health [23], including the anticancer [30], antihypertensive [31], antihyperlipidemic [32], anti-inflammatory [33], and antioxidant [34] effects of kefir.

Researchers have demonstrated the significant antioxidant activity of bacteria isolated from milk kefir, as well as the subsequent therapeutic properties of this beverage [35]. However, a large proportion of consumers has excluded dairy products from their daily diet, either due to medical reasons, such as allergies or intolerance to them, or due to the adoption of an exclusively plant-based diet (vegan diet) [36]. An alternative source of antioxidants and other healthy bioactives for such consumers, who cannot enjoy the health benefits of kefir-fermented dairy, is water kefir [25]. Non-dairy water kefir-derived beverages show significant antioxidant activities and other health benefits, which are attributed to the symbiotic microorganisms present in kefir [34],[37].

The microflora composition of water kefir was studied by many researchers during 1980-2011, with some bacteria, such as *Lactobacillus*, and yeasts, such as *Saccharomyces cerevisiae*, being repeatedly observed [38], while metagenomic analysis revealed that other bacteria, like the ethanologenic bacterium *Z. mobilis*, are also abundant, both in water kefir grains and in the final fermented product [26]. Many commercial water kefir grains contain other species of bacteria, belonging to the genera *Acetobacter, Bifidobacterium, Leuconostoc*, and *Lactococcus lactis* and various species of fungi, classified under the genera *Dekkera*, *Hanseniaspora*, *Lachancea*, *Zygosaccharomyces*, and *Zygotorulaspora* [39]. The qualitative and quantitative composition of the microorganisms present in kefir depends on several parameters, including the season and the area where the grains are collected, as well as the specific process used for the preparation of the fermented water kefir product (i.e., beverage) [40].

Here, we present the most commonly founds in water kefir microorganisms and their bioactive metabolites that have been reported to exhibit several health-promoting effects, with emphasis on their antioxidant and anti-inflammatory bio-functionalities with associated health benefits.

### 2.1. Water kefir lactic acid bacteria and their bioactive metabolites

#### 2.1.1. Lactobacillus casei

*Lactobacillus casei* is one of the main lactic acid bacteria (LAB) found in water kefir [41], and has been widely studied as a probiotic used to ferment various other products, such as cheese, with health-promoting effects [43]. For example, fermentation for producing mature cheeses by monocultures of *L. casei* and *Propionibacterium* altered the proportion of saturated and unsaturated fatty acids beneficially and thus reduced the values of atherogenic and thrombogenic indexes, potentially inducing important related health benefits [42]. Moreover, fermentation of *Gymnema sylvestre* leaves with *L. casei* resulted in probiotic-based fermentative conversion of gymnemic acid-enriched *G. sylvestre* leaf extract to gymnemagenin-containing nutraceuticals since gymnemagenin is the bioactive metabolite of this plant that possesses a different therapeutic potential for diabetes and other metabolic disorders, including its use in ayurvedic and homeopathic frameworks of medication, as well as against asthma, breathing disorders, chronic cough, colic pain, constipation, dyspepsia, eye complaints, family planning, heart disease, hemorrhoids, hepatosplenomegaly, piles, stomach ailments, snakebite, and urinary complaints; it also possesses antimicrobial, antitumor, anti-obesity, anti-hyperglycemic and anti-inflammatory activity [44]. Production of several fermented products by fermentation based on *L. casei* as a probiotic resulted in increased phenolic compounds and carotenoids [45],[46] and a significant increase in the bioactive metabolites and their antioxidant activity [43],[47].

LABs, including *L. casei*, have been proposed to provide several health benefits when administered as probiotics. For example, they have been studied as a probiotic treatment for disturbed microbiota in patients with chronic fatigue syndrome [48]. Studies in animal models revealed that LABs and their metabolites can potently prevent immune-modulating and inflammatory processes, for example, by increasing dendritic and regulatory T cells as well as decreasing the levels of inflammatory cytokines like tumor necrosis factor alpha (TNF-α). Moreover, human trials with probiotics, including *L. casei* and *Bifidobacterium infantis*, showed a decrease in pro-inflammatory levels in the probiotic intervention groups [48]. Another placebo-controlled randomized clinical trial (RCT) demonstrated a dose-dependent amelioration of perceived fatigue after 8 weeks of *Lactobacillus* and *Bifidobacterium* supplementation in patients with irritable bowel syndrome (IBS) [49].

Beneficial modulation of the gut microbiome and protective roles of a bioactive *L. casei* strain, *L. casei*^+mcra^, have also been reported since the inserted mcra (myosin cross-reactive antigen) gene in this strain stimulates the conversion of conjugated linoleic acids, especially against pathogenic *Salmonella enteric serovar Typhimurium* and enterohemorrhagic *E. coli* infections in mice. *L. casei*^+mcra^ colonized the pathogen-challenged mice gut intestine efficiently and competitively reduced the infection with these pathogens in various locations of the small and large intestines, while it also showed a positive modulation of the cecal microbiota and increased bacterial species diversity/richness. Moreover, a subsequent attenuation of bacterial pathogen-induced gut inflammation was also observed by reducing the expression of pro-inflammatory cytokines, suggesting that daily consumption of novel probiotics that over-produce conjugated linoleic acids effectively improves intestinal microbiota composition and prevents/combats foodborne enteric bacterial infections with pathogenic *Salmonella* and diarrheagenic *E. coli* [50].

#### 2.1.2. Lactobacillus hilgardii

*Lactobacillus hilgardii* is one of the LAB strains of water kefir [51] and of other fermented food products, including wine [52]. Most strains produce exopolysaccharides (EPS) in water kefir [51], which can be considered as natural bio-thickeners as they are produced *in situ* by such LAB species and improve the rheological properties of fermented foods, while EPS from LABs have shown beneficial effects on modulating the gut microbiome and thus promoting health, as well as several other diverse health effects, such as glycemic control, calcium and magnesium absorption, cholesterol-lowering, anticarcinogenic, immunomodulatory, and antioxidant effects [53].

Generally, the bacteria were found in products that showed antioxidant [52],[54] and anti-inflammatory [54] capabilities. For example, *L. hilgardii* can strongly inhibit lipopolysaccharide (LPS)-induced secretion of inflammatory cytokines like TNF-α, interleukin (IL)-1β, and IL-6 in mouse splenocytes [55]. Moreover, fermentation of *Sesbania cannabina* by *L. hilgardii* and other LAB resulted in the production of anti-inflammatory compounds, such as psoralidin and alnustone, as well as antioxidant compounds like lithospermic acid [56]. *L. hilgardii*, as a probiotic, also improved blood glucose and blood lipid metabolism and pancreas function by increasing the expression of gut-secreted hormones with anti-obesity and anti-diabetic actions, such as the glucagon-like peptide-1 (GLP-1) and peptide YY (PYY) [54]. Such LAB strains in the wine were able to respond to phenolic acids by increasing unsaturated fatty acids [57]. Nevertheless, phenolic acids show a negative effect on the growth of *L. hilgardii*, with the exception of gallic acid [58].

#### 2.1.3. Lactobacillus hordei

*Lactobacillus hordei* also belongs to the LAB species of water kefir [59]. *L. hordei* strains ferment acidic, high-sugar media like water kefir or fruit juices, which are otherwise poor in nitrogen sources and produce dextrans from sucrose. Furthermore, it is speculated that *L. hordei* produces ammonia upon arginine hydrolysis to protect itself from pH stress during alkalization of its cytoplasm and proximal environment and that it reduces external acid stress by switching from lactate and acetate production to butanediol formation [60]. During the growth of *L. hordei* in water kefir, alternative enzyme functions were utilized for the biosynthesis of unsaturated fatty acids (UFA) [59]. UFAs, which are a key part of the Mediterranean diet, have been reported for their positive effect on health, including their anti-inflammatory activity [61].

#### 2.1.4. Lactobacillus kefiri

*Lactobacillus kefiri*, which is a LAB isolated from kefir, has shown potential for immunomodulatory response activity in several studies. In some cases, *L. kefiri* treatment resulted in an increase in the expression of IL-10 in mesenteric lymph nodes through the pro-inflammatory cytokines IL-23, interferon gamma (IFN-γ), and IL-6 [62]. Similarly, *in vivo* studies in mice showed that such a treatment suppressed the expression of IL-6 and granulocyte macrophage colony stimulating factor (GM-CSF) in ileum and colon explants, while IL-10 expression was increased in colon explants, suggesting a potential anti-inflammatory activity of *L. kefiri* [62].

Additionally, fermented milk containing *L. kefiri* showed anti-inflammatory effects *in vivo* in mice with periodontitis inflammation, since TNF-α, IL-1β, and IL-6 were reduced and IL-10 increased [63]. *L. kefiri* can also beneficially modulate the gut microbiota composition and thus promote health against gastrointestinal diseases by inducing a reduction of bacteria with pro-inflammatory activity and by contributing with anti-inflammatory effects [64]. Finally, one of the exopolysaccarides produced by *L.kefiri* (MSR101 EPS) showed antitumor activity against colon cancer HT-29, as it induced an increase in the expression of signaling molecules with antitumor activities, such as BCL2 associated agonist of cell death (BAD) protein, caspase 3, caspase 8, caspase 9, and cytochrome-c, but also a decrease in the expression of tumor-inducing molecules, like B-cell lympoma 2 [65].

#### 2.1.5. Lactococcus lactis

*Lactococcus lactis* has been used historically in fermentation and food preservation processes as it is considered safe for human consumption (GRAS, Generally Recognized As Safe); while it has also been detected as one of the most common MO in water kefir [66] and in other fermented food sources like ricotta cheese [67]. It has a wide temperature range for growth and tolerance in challenging conditions such as high osmotic concentrations, acidity and bile salts, alkaline conditions, and heat and cold shock membrane stress [67],[68].

Many probiotic bacteria have been described as promising tools for the treatment and prevention of inflammatory bowel diseases (IBDs). Most of these bacteria are LAB, which are part of the healthy human microbiota. However, the effects of transient bacteria in normal diets, including *Lactococcus lactis*, have recently been evaluated. Several studies have outlined several bioactivities for several strains of this LAB and its metabolites, based on both *in vivo* and *in vitro* assessments. For example, *L. lactis* has presented probiotic characteristics, antioxidant and inhibitory capacity for lipid peroxidation, and compatible safety aspects for use as a food technology culture [67]. Similar health-promoting effects, including anti-hypertensive effects like the inhibition of the angiotensin-converting enzyme (ACE), antioxidant capacity, and antidiabetic activity like the inhibition of the glucosidase and amylase enzyme activities, have also been reported for fermented foods produced by fermentations based on the probiotic *L. lactis* [69].

Administration of specific strains of *L. lactis* alleviated colitis induced by dextran sulfate sodium (DSS) in mice through the inhibition of inflammatory cell infiltration, as it significantly ameliorated shortening of colon length and histological score of the colon, while it also improved the aberrant mRNA expression in inflamed tissue close to the control level through notable suppression of the mRNA expression of pro-inflammatory cytokines and mediators, including TNF-α, IL-6 and IFN-γ, NO-synthases (iNOS), and Macrophage inflammatory protein-2 (MIP-2) [70]. Moreover, treatment with the same strains of *L. lactis* in an *in vitro* inflammatory co-culture system consisting of intestinal epithelial Caco-2 cells and RAW264.7 macrophage cells (stimulated by LPS) resulted in significant down-regulation of IL-8 mRNA expression in Caco-2 cells and inhibition of NF-κB nuclear translocation in RAW264.7 cells [70]. Similarly, other strains of *L. lactis* also showed a potential role in the treatment of IBD by an anti-inflammatory effect, as they were able to reduce IL-1β-induced IL-8 secretion *in vitro* in Caco-2 cells, suggesting a potential anti-inflammatory effect, while oral treatment with this LAB resulted in a milder form of recurrent colitis than that observed in control diseased mice [71].

The anti-inflammatory potential of bioactive peptides isolated from several strains of *L. lactis* has also been reported. For example, two *L. lactis* active peptides dose-dependently inhibited LPS-induced inflammatory cytokines TNF-α, IL-6, and IL-1β and inflammatory factors NO and PGE 2 production in macrophages. These *L. lactis*-derived peptides also blocked the expression of Toll-like receptor 2 (TLR2) and then suppressed the phosphorylation of NF-κB, p65, and the degradation inhibitor of nuclear factor of kappa light polypeptide gene enhancer in B-cells inhibitor alpha; this sugests that their anti-inflammatory properties might be a result of the inhibition of IL-6, IL-1β, and TNF-α expressions through the downregulation of Toll2/NF-κB signaling pathways, relieving pathological inflammatory responses caused by LPS-induced inflammatory responses *in vivo* in *Ctenopharyngodon idella* [72]. Moreover, by editing the proteolytic system of *L. lactis*, both the number of different bioactive peptides and the bioactivity diversity can be increased for this LAB, with a clearly strain-dependent accumulation of peptides with several health-promoting bioactivities, such as antioxidant activities (dipeptidyl peptidase 4 inhibition), anti-hypertensive effects (ACE-inhibition), and immunoregulatory functions [73].

In addition, fermentation of food sources like milk by specific strains of *L. lactis* facilitates the development of fermented functional foods rich in potent bioactive peptides with various biological activities that may have a positive effect on cardiovascular health, such as antithrombotic and hypocholesterolemic activities, including inhibition of thrombin-induced fibrin polymerization, anticoagulant activity, inhibition of micellar solubility of cholesterol, and bile acid binding capacity of water-soluble fractions [74].

The presence of LAB probiotics like *L. lactis* in the gut microbiota is also responsible for differential anticancer drug efficacy by modulating the host immune system and the tumor microenvironment, while this differential effect is highly strain-specific. For example, certain gut microbiota strains can directly suppress tumor growth and enhance antitumor immunity while others do not have such an effect or promote tumor growth. Identifying effective strains with antitumor effects is important for developing live biotherapeutic anticancer products. Interestingly, a strain of *L. lactis* is a promising candidate for potentiating cancer treatment in combination with current standard therapy as it was found to inhibit tumor growth by regulating tumor angiogenesis and directly inducing cancer cell death; moreover, it can enhance the therapeutic effects of oxaliplatin and the PD-1 blockade, by augmenting cytotoxic immune cell populations, such as CD4+ T cells, CD8+ effector T cells, and NK cells in the tumor microenvironment [75].

In addition, *L. lactis* has also been successfully used as a bioreactor with gene expression systems known as “food-grade systems” for the production of molecules of medical interest and safe selection markers, as well as vectors for DNA delivery, suggesting new biotechnological and therapeutic uses of *L. lactis* [76]. Moreover, recombinant strains of this LAB have been successfully used as safe mucosal delivery of DNA expression vectors that code for anti-inflammatory compounds like the IL-10 cytokine. For example, the development of a Stress-Inducible Controlled Expression (SICE) system in *L. lactis* for the production and delivery of proteins of health interest (both therapeutic and vaccine-related) at mucosal surfaces has also been reported, as validated *in vivo* in a model of therapy against IBD and a model of vaccination against human papillomavirus type-16 (HPV-16) [77]. The facilitation of the *in situ* delivery of the anti-inflammatory IL-10 cytokine by the use of genetically engineered strains of *L. lactis* seems to be efficient as a therapy to treat low-grade colon inflammation [78] and food-induced IgE sensitization [79], as it attenuated intestinal inflammation in IL-10-deficient mice [80] and reduced inflammation in a murine model of experimental colitis [81].

Similarly, preventative delivery of other anti-inflammatory cytokines like IL-35 by engineered *L. lactis* strains could ameliorate DSS-induced ulcerative colitis in mice by inducing an anti-inflammatory potential as observed by the increased colon and serum levels of IL-10 with a concomitant reduction of pro-inflammatory cytokines like IL-6, IL-17A, IFN-γ, and TNF-α [82].

Finally, intranasal immunization with a recombinant *L. lactis* strain secreting murine IL-12 (a cytokine with an important role in cellular immunity to several bacterial, viral, and parasitic infections, which has also been used as adjuvant activity when co-delivered with DNA vaccines, as well as with success in cancer immunotherapy treatments) could enhance antigen-specific Th1 cytokine production and thus enhance an antigen-specific immune response and stimulate local mucosal immunity [83].

#### 2.1.6. Lactobacillus mali

*Lactobacillus mali* is a LAB isolated from water kefir [41] and is used to ferment several other products, like pumpkin juice. The fermentation of pumpkin juice by *L. mali* revealed an elevation in dietary phenolics and antioxidant capacity, as shown in the ferric reducing antioxidant power (FRAP) and 2,2-diphenyl-1-picrylhydrazyl (DPPH) free radical scavenging activity assays. Due to the higher content of phenolics and flavonoids, an antidiabetic potential was also induced, such as the reduction of the IC50 values against α-amylase and α-glucosidase, and thus an increase in the inhibitory effects against both of these enzyme activities [84].

*L. mali* strain APS1 induced metabolic changes *in vivo* in rats that led to the production of butyrate and GLP-1, which activated the expression of Sirtuin-1 (SIRT-1), which further induced nuclear erythroid 2-related factor 2 (Nrf2) expression and activation, leading to suppression of hepatic oxidative stress through the increasing production of antioxidant enzymes in the liver [85], like heme oxygenase-1 (HO-1). Another study highlighted the ability of *L. mali* APS1 to suppress inflammatory responses via inhibiting inflammatory cytokines and enhancing regulatory T cells (Treg) cytokines [86]. *L. mali* was also able to significantly induce IL-8 secretion from human colorectal adenocarcinoma cell lines with epithelial morphology (HT-29), thus suggesting anti-inflammatory capabilities [87]. Additionally, APS1 upregulated M2 activating macrophages in high-fat diet (HFD)-induced obese mice by significantly downregulating relative mRNA expressions of M1 macrophages and M1 activating markers (macrophage inflammatory protein MIP-1, Interleukin-1 receptor antagonist IL-1ra. Thus, this study suggested a link between probiotic intervention, obesity, and inflammation [88].

#### 2.1.7. Lactobacillus nagelii

*Lactobacillus nagelii* is another LAB that has been detected and studied in water kefir [41] and is in other fermented foods such as kombucha. The inherent capability of *L. nagelii* is to metabolize glucose into several products, including several bioactive metabolites, such as vitamin B compounds [89], fatty acids [60], and polyphenols [90]. In an *in*-*vivo* study, oral administration of bretanin and *L. nagelii* cells in mice resulted in an improved lipid profile and a beneficial change in blood glucose [91]. Moreover, *L. nagelii* has been observed in products with hypotensive, hypoglycemic, and cholesterol-lowering activities [90] and antioxidant properties, which correlates with an increase in their polyphenol content [92]. Moreover, the flagellin proteins of *L. nagelii* also induced IL-8 secretion from HT-29 cells, which indicates potential anti-inflammatory activity [87].

#### 2.1.8. Lacticaseibacillus paracasei

*Lacticaseibacillus paracasei*, also known as *Lactobacillus paracasei*, is a LAB that belongs in the *Lactobacillus casei* Group and has been isolated from different sources, including water kefir, while it is used to ferment juices and traditional Korean food products. Fermentation of pomegranate juice by *L. paracasei* enhanced the antioxidant activity of the final fermented product [93], with a simultaneous increase in the total phenolic content of the beverage due to the transformation of the phenolic compounds contained in the juice by enzymes involved in the metabolic activities of *L. paracasei* strains.

*L. paracasei* has also been used as a probiotic in okara beverage, where it demonstrated an increase in total phenols and flavonoids and a potential for efficient enrichment of bioactive isoflavone glycosides because of its high acid production rate. The *L. paracasei* group showed the highest antioxidant capacity among all the samples [94]. Additionally, the *L. paracasei* NSMJ56 strain enhanced the percentage of T cell subpopulations, particularly CD4+ T cells. The role of this strain as a dietary probiotic in gut health via shaping gut microbiota and local immunity has also been explored in laying hens [95]. Fermented soy germ with the *L. paracasei* DCF0429 strain displayed strong antioxidant abilities. The study showed that the soy germ-based postbiotic SGPB-DCF0429 had a cytoprotective effect, significantly reducing H2O2-induced TNF-α levels. It also downregulated IL-6 and IL-8 in a dose-dependent manner, mirroring the reduction in TNF-α. SGPB-DCF0429 consistently inhibited the expression of TNF-α, IL-6, and IL-8 in H2O2-stimulated Keraskin, showcasing its ability to protect human epidermal tissues from oxidative stress through anti-inflammatory mechanisms. This highlights the potential of SGPB-DCF0429 as a novel cosmetic ingredient with protective and anti-inflammatory properties [96].

Moreover, the *L. paracasei* M7 strain produced EPS that exhibited multifaceted biological activities, including high *in vitro* DPPH antioxidant scavenging activity and hypocholesterolemic (cholesterol-lowering) effects [97]. Similarly, in the specific *L. paracasei* DG strain, two gene clusters putatively coding for EPS biosynthesis were identified, and thus it was demonstrated that it can produce a unique rhamnose-rich hetero-exopolysaccharide named DG-EPS, which displays immune-stimulatory properties by enhancing the gene expression of the pro-inflammatory cytokines TNF-α and IL-6 and, particularly, the chemokines IL-8 and chemokine ligand 20 (CCL20) [98].

#### 2.1.9. Leuconostoc citreum

The LAB *Leuconostoc citreum* is also part of the MO of water kefir [38] and has been isolated from several other sources. It has the ability to tolerate stress, have significant resistance capacities from gastric juice, and endure harsh conditions of temperature, oxidative factors, acid exposure, bile salts, and proteolytic enzymes [99]. *L. citreum* is widely used in a variety of biological applications while it produces several bioactive metabolites by being able to ferment and metabolize many carbohydrates [99]. For example, treatment of 3T3-L1 adipocytes with cell-free metabolites of *L. citreum* (LSC) reduced the accumulation of lipid droplets and lipogenesis, elevated the levels of adiponectin (an insulin sensitizer), and increased lipolysis, suggesting that *L. citreum* is a probiotic strain with health-promoting properties against obesity and its associated metabolic disorders [100].

*L. citreum* isolated from malted barley can produce bacteriocins, which are peptides with antibacterial properties that affect genetically closely related bacteria, suggesting that when bacteriocin-producing probiotics are settled in the GI tract, they can contribute to bacteriocin formation, which can lead to pathogen inhibition and immune system regulation. Moreover, *L. citreum* strains have also shown significant probiotic potential and strong antibacterial activities against different pathogens *in vitro*, suggesting that these strains could be used instead of antibiotics to control infectious pathogens [101]. In addition, such heat-killed bacteriocin-producing strains suppressed the mRNA expression levels of inflammatory cytokines and chemokines in RAW 264.7 macrophages induced by LPS, thus exhibiting anti-inflammatory effects too [99].

*L. citreum* isolated from Korean kimchi was appropriately engineered to be able to convert isoflavone glycosides present in fermented soymilk into their aglycone soy isoflavone forms in order to increase their bioavailability, as the aglycon forms of these bioactive phytochemicals, with anti-aging, anti-tumor, and antioxidant properties, are more efficiently absorbed through the gut epithelium [102]. After fermentation of optimized soybean whey-enriched 10% sucrose at 37 °C for 24 h with *L. citreum* resulted in the synthesis of an EPS from this MO, which exhibited certain antioxidant capacity through scavenging activity on the ABTS radical [103], while another EPS produced by *L. citreum* also showed strong antioxidant potential as assessed for its scavenging potential in the DPPH assay, as well as antioxidant effects against hydroxyl (·OH), superoxide anion (O^2−^), hydrogen peroxide (H_2_O_2_), and nitroso radical (NO^2−^) scavenging abilities, and reducing power [104].

Finally, *L. citreum*, isolated from chive, produces glucansucrase and synthesizes bioactive oligosaccharides through its enzymatic activity, which exhibited a prebiotic effect on six bacterial and yeast strains, while also showing anti-inflammatory activity in RAW 264.7 macrophage cells [105]. More specifically, in LPS-stimulated RAW 264.7 cells treated with these gluco-oligosaccharides from *L. citreum*, the production of NO was decreased, the expression of iNOS, TNF-α, IL-1β, IL-6, and IL-10 was suppressed, and the NFkB signaling pathway was inhibited, resulting in an overall anti-inflammatory effect.

#### 2.1.10. Leuconostoc mesenteroides

The LAB *Leuconostoc mesenteroides* has been detected in kefir grains [106] and its health-promoting effects as a probiotic have been studied against several inflammatory disorders and infections in the GI tract, as well as in other cavities with microflora, like the oral cavity. For example, *L. mesenteroides* exerts anti-inflammatory activity to maintain oral health, as it exhibited beneficial probiotic anti-inflammatory health-promoting effects against periodontitis, a chronic inflammatory infectious oral disease caused by *Porphyromonas gingivalis* [107]. More specifically, *L. mesenteroides* effectively showed oral tissue adherence and colonization abilities to gingival epithelial and HT-29 cells, as it inhibited *P. gingivalis* biofilm formation and *P. gingivalis-*induced oral inflammation in an *in vitro P. Gingivalis* LPS-stimulated RAW 264.7 macrophage cell-based inflammation model, by reducing the secretion of pro-inflammatory cytokines (TNF-α, IL-6, and IL-1β) and inflammatory mediators (NO and PGE2), and decreasing the expression levels of inflammation-related genes.

Intracellular extracts of *L. mesenteroides* have also shown a higher potential to provide some levels of host protection against porcine epidemic diarrhea virus (PEDV) infections in a Vero cell culture model of infection in comparison to extracts from other kefir microorganisms assessed. The extracts of *L. mesenteroides* possessed *in vitro* prophylactic, therapeutic, and direct-inhibitory effects against PEDV in this cell model, by up-regulating the expression of Type 1 IFN-dependent genes, including Myxovirus resistance 1 (MX1) and INF-stimulated gene 15 (ISG15), after treatment with intracellular extracts of a specific *L. mesenteroides* strain for 24 h.

*L. mesenteroides* has been utilized to produce bioactive fermented products as the main fermenting microorganism. For example, oral administration of *L. mesenteroides* isolated from kimchi, a fermented food rich in probiotics, exhibited anti-inflammatory health-promoting properties as a probiotic by alleviating ulcerative colitis by improving the inflammatory response and mucosal barrier function in the colon of mice [108]. More specifically, reduced symptoms of colitis caused by DSS, such as disease activity index, decrease in colon length, colon weight-to-length ratio, and pathological damage to the colon, were observed, while decreased levels of pro-inflammatory cytokine TNF-α and increased anti-inflammatory cytokine IL-10, as well as reduced mRNA and protein expression levels of inflammatory factors in the colon tissues, were also found.

Moreover, *L. mesenteroides* isolated from Chinese pickled cabbage (Sichuan paocai) produces a released exopolysaccharide (rEPS) with strong *in vitro* antioxidant activity, cholesterol-lowering properties, and potential antitumor activity [109], suggesting that this microorganism and its rEPS are promising probiotics with broad health-promoting prospects in industry.

*L. mesenteroides* can utilize several carbon sources to produce bioactive EPS, with sucrose resulting in the highest yield in *L. mesenteroides* isolated from Dajiang, a traditional fermented food in northeast China [110], while the EPS produced from such a fermentation showed higher antioxidant activity (hydroxyl radical scavenging rate and DPPH scavenging rate) compared to that of vitamin C under the same concentrations. In addition, a levan-type EPS, produced by a strain of *Leuconostoc mesenteroides*, showed a high level of immune-modulatory role, induced the anti-inflammatory cytokine IL-4, and exhibited a strong in vitro antioxidant capacity (hydroxyl radical scavenging activity, compared to ascorbic acid), suggesting the potential of this levan (S81) for technological purposes and as a potential natural immunomodulatory and antioxidant bioactive, with potential antitumor activity too [111].

In comparison with non-fermented samples (control), the fermentation for 24 h of hydroponic ginseng (HPG) roots and shoots with *L. mesenteroides* isolated from kimchi resulted in increased total phenolic and flavonoid contents and enhanced antioxidant activity, as assessed by ABTS, β-carotene-linoleic, and FRAP assays, as well as increased anti-inflammatory effects, by reducing the nitric oxide content and the expression of inducible iNOS, TNF-α, IL-1β, and IL-6 in LPS-stimulated RAW 264.7 macrophages treated with fermented HPG, and enhanced anti-adipogenic effects by reducing the lipid accumulation in 3T3-L1 adipocytes when treated with fermented HPG [112]. These findings further suggest that fermented products by *L. mesenteroides*, such as the fermented HPG, have potential for health-promoting applications in the functional food industry.

In addition, fermentation of a garlic-Cirsium setidens Nakai blend based on *L. mesenteroides* resulted in increased levels of produced phenolics (polyphenols and phenolic acids) and flavonoid compounds, while such a fermentation also increased the levels and release of anti-inflammatory compounds, like pectolinarin and pectolinarigenin, and thus increased the bioavailability of such not so bioavailable bioactives [113]. The FRAP ferric reducing ability and DPPH radical scavenging activities of all the fermented samples assessed increased significantly after fermentation, while their ethanolic extracts significantly enhanced RAW 264.7 macrophage proliferation and induced the production of nitric oxide and pro-inflammatory cytokines TNF-α and IL-1β, as well as an anti-inflammatory cytokine IL-10, suggesting that extracts of these fermented products harmonize the interplay of proinflammatory factors and anti-inflammatory cytokines that facilitates the appropriate resolution of infections.

Finally, dextransucrase from *L. mesenteroides* catalyzes the synthesis of two glucosides of caffeic acid phenethyl ester (CAPE) and its bioactive derivatives [114], which possess several pharmaceutical properties including antimicrobial, antioxidant, anti-inflammatory, and cytotoxic effects [115]. Compared to CAPE, the monoglycoside product showed superior anti-inflammatory effects, with potent inhibitory effects against NO, IL-6, and TNF-α in RAW 264.7 macrophages, at micromolar concentrations. Also, the cytotoxicity of both glucoside-CAPE products was significantly improved. These glycosylation-modified CAPEs circumvent some of the flaws in CAPE application as anti-inflammatory pharmaceuticals. Moreover, the synthesis of other bioactive glycosylated products by the enzymatic activity of dextransucrase from *L. mesenteroides* has also been reported, such as the production of novel ampelopsin glucosides (AMPLS-Gs), which showed competitive inhibition against tyrosinase that was comparable to the same effect observed in commercial active ingredients of whitening cosmetics like β-arbutin, indicating the potential of AMPLS and AMPLS-G1 as superior ingredients for functional cosmetics [114],[116].

#### 2.1.11. Oenococcus Oeni

*Oenococcus oeni*, also known as *Leukonostok oeni*, is another LAB species present in water kefir [51] that is most frequently associated with malolactic fermentation (MLF), especially in wine [117].

Several *O. oeni* strains have exhibited antioxidant activities [118], with widely dispersed antioxidative parameters assessed, irrespective of the evaluation methods used, which indicated that antioxidative properties depended on the strain and culture medium. *O. oeni* strains showed antioxidant mechanisms assigned to their DPPH scavenging ability, ROS scavenging ability, iron chelation (FE), glutathione system, FRAP, reduction activity (RA), inhibition of ascorbic oxidation (TAA), and linoleic acid oxidation (TLA) abilities. Most of the *O. oeni* strains possess promising potential probiotic characteristics, serving as defensive agents in the intestinal microbial ecosystem and overcoming exogenous and endogenous oxidative stress, while they also exhibited good survival abilities in low pH values (pH 1.8), simulated intestine juice and bile salts (1%), suggesting their good adaptation to gastrointestinal conditions and high bile resistance abilities.

Fermentation of sterile grape juice (SGJ, pH 4.0) by *O. oeni* strains increased the antioxidant activity of this fermented product without however affecting or even decreasing its total phenolic content, suggesting that the antioxidant effect seems to be attributed to other bioactives, while the *O. oeni* strains used in this fermentation also showed antibacterial properties against *E. coli*, *Salmonella Typhimurium* and *Listeria monocytogenes* [119]. Similarly, the MLF conducted by *O. oeni* in ciders enables the increase of interesting biological activities and functional properties in this fermented product since strains of this MLF LAB in apple cider fermentations increased the antihypertensive effects (increased angiotensin I-converting enzyme inhibition) and antioxidant activities (increased FRAP and ABTS radical-scavenging activities) of the apple cider product [120], along with modifications in phenolic compounds and nitrogen organic compounds after the malolactic fermentation.

Apart from the widely used probiotic bacteria, *Lactobacillus* and *Bifidobacterium*, of the human gastrointestinal tract, other “less conventional” bacteria, from allochthonous or extremophilic origin, sharing similar structural or functional features, may also confer specific health benefits to a host, such as the *O. oeni* MLF LAB, which has exhibited immunomodulatory or immune-stimulatory activities by inducing strain-specific cytokine patterns released by peripheral blood mononuclear cells (PBMCs) [121]. *O. oeni* bacteria strains showed also *in vivo* anti-inflammatory potential in an experimental 2,4,6-trinitrobenzene sulfonic acid (TNBS)-induced colitis mouse model, in which a specific probiotic strain significantly lowered colonic injury and alleviated colitis symptoms. These potential immunomodulatory health-promoting effects of *O. oeni*, combined with their ‘natural’ tolerance towards acid, ethanol, and phenolic compounds, suggest the possibility of selected *O. oeni* strains being used as live probiotics.

**Table 1.** Bioactives and health promoting properties of water kefir LAB microorganisms.

LAB Microorganism	Bioactive(s) compound	Health promoting effect	Ref
*Lactobacillus casei*	Fermentation of Gymnem sylvestre leaves with the MO	Gymnemagin nutraceuticals, potential for improvement of diabetes and several metabolic disorders, antimicrobial, antitumor, anti-obesity, anti–hyperglycemin and anti-inflammatory activity	[44]
	Fermented products	Probiotic activity	[48]
	The MO itself	Increase of phenolic compounds	[45]
		Carotenoids increase	[46]
		Antioxidant activity and bioactive metabolites production increase	[43],[47]
	The MO itself as probiotic	Probiotic activity for chronic fatigue syndrome	[48],[49]
	Conjugated linoleic acids of the MO	Protective against salmonella and *E. coli* and gut microbiota composition, pro-inflammatory cytokine reduction	[50]
	The MO with Propioni bacterium	Atherogenic and thrombogenic indices reduction	[42]
*Lactobacillus hilgardii*	EPS produced	Glycemic control, cholesterol lowering, anticarcinogenic, immunomodulatory and antioxidant effects	[51],[53]
	LPS-induced inhibitors for *L. hilgardii*	TNF-α, IL-1β, IL-6 secretion	[55]
	The MO itself as probiotic	Glucose and lipid metabolism enhancement. pancreas function improvement (with GLP-1 and YY peptide)	[54]
	Fermentation of Sesbania cannabina with *L. hilgardii* and other LAB	Anti-inflammatory compounds production (psoraldin and alnustone), antioxidant compound production (lithospermic acid)	[56]
*Lactobacillus hordei*	The MO with other LAB strains	Antioxidant perspectives	[45]
*Lactobacillus kefiri*	The MO itself	Pro-inflammatory, anti-inflammatory activity	[62]
	Fermented milk with *L. kefiri*	Periodontitis inflammation reduction	[63]
	MO strain	Gut microbiota modulation, effective against gastrointestinal diseases	[64]
	MSR101 EPS produced	Antitumor activity	[65]
*Lactobacillus lactis*	*L. lactis* strains	pro- and inflammatory activity with cytokine response production (TNF–α, IL-6, IFN–γ, INOS, MIP-2)	[70]
	*L. lactis strains*	IBD treatment (IL-1β reduction and IL-8 secretion)	[71]
	Peptides of *L. lactis*	Inflammatory activity with cytokine inhibition (IL-1β, TNF–α, IL-6)	[72]
	Proteolytic system of *L. lactis*	Antioxidant effects and anticoagulant immunoregulatory action	[73]
	Strains of *L. lactis* in gut microbiota	Antitumor activity and potential anticancer activity	[75]
	Engineered *L. lactis* strains	Anti-inflammatory activity with cytokine IL-35, DSS-induced colitis improvement, reduction of pro inflammatory cytokines	[82]
	Recombinant *L. lactis*	Promote of Th1 cytokine production, potential anticancer use	[83]
*Lactobacillus mali*	Phenolic acids	Increased dietary antioxidant contents in fermented pumpkin juice	[84]
	Flavonoids	Enhanced antioxidant activity (FRAP, DPPH)	[84]
	Butyrate	Induced metabolic changes, potentially beneficial for gut health	[85]
	GLP-1	Activation of SIRT-1 expression and subsequent antioxidant effects	[85]
	SIRT-1	Suppression of hepatic oxidative stress through Nrf2 activation	[85]
	Nrf2	Upregulation of antioxidant enzymes in the liver	[85]
	Heme oxygenase-1 (HO-1)	Increased hepatic antioxidant activity	[85]
	MO itself	Suppression of inflammatory cytokines	[86]
		Enhancement of Treg cytokines	[86]
	IL-8 secretion	Suggested anti-inflammatory capabilities	[87]
	Modulation of M1/M2 macrophages	Regulation of obesity-associated inflammation	[88]
*Lactobacillus nagelii*	Bretanin and *L. nagelii* strains in mice	Enhanced lipid profile and alteration in blood glucose levels	[91]
	MO in products	Observed in products with hypertensive, hypoglycemic, cholesterol-lowering activity (may contribute but further research has to evaluate that)	[90]
	MO observed in products	Antioxidant action (polyphenols increase)	[122],[123]
	Flagellin proteins of *L. nagelii*	IL-8 expression reduction, potential anti-inflammatory activity	[87]
*Leuconostoc mesenteroides*	The MO itself as probiotic	Oral probiotic anti-inflammatory health promoting effects against Periodontitis	[107]
	The MO itself as probiotic	Probiotic benefits as it alleviates ulcerative colitis by improving the inflammatory response and mucosal barrier function in the colon	[108]
	intracellular extracts	host protection against porcine epidemic diarrhea virus infections	[106]
	EPS	Anti-oxidant, anti-inflammatory, antitumor effects, with health promoting prospects in several industry applications	[111]
	Fermentation of HPG with *L. mesenteroides*	Functional food product with increased total phenolic and flavonoid contents, enhanced antioxidant activity, increased anti-inflammatory effects and enhanced anti-adipogenic properties	[112]
	Fermentation of garlic with *L. mesenteroides*– Ethanolic extracts	Functional food product with increased total phenolic and flavonoid contents, enhanced antioxidant activity, increased levels and release of the anti-inflammatory pectolinarin and pectolinarigenin, modulation of inflammatory cell profile for anti-infectious effects	[113]
	glucosides of caffeic acid phenethyl ester produced by *L. mesenteroides* dextransucrase	Improved anti-inflammatory pharmaceutical properties	[114]
	ampelopsin glucosides produced by *L. mesenteroides* dextransucrase	Whitening effect (competitive inhibition against tyrosinase) for functional cosmetics	[116]
*Leuconostoc citreum*	Metabolites of *L. citreum*	Obesity and metabolic disorders treatment	[80]
	Bacteriocin producing strains of *L. citreum*	Antibacterial and anti-inflammatory activity (inflammatory cytokines and chemokines reduction)	[99]
	Engineered *L. citreum*	Anti-aging, anti-tumor, antioxidant properties (isoflavone bioactives)	[102]
	EPS produced	Antioxidant activity	[103]
	EPS produced	Antioxidant activity	[104]
	Glucansucrase, glucooligosaccharides produced	Prebiotic and anti-inflammatory action	[105]
*Oenococcus oeni*	MO strains	Antioxidant activity	[118]
	MO strains	Antioxidant and Antibacterial against *E. coli*, *salmonella*, *typhimurium* and *listeria monocytogenes*	[119]
	MLF conducted by *O. oeni* in ciders	Antihypertensive and antioxidant effect (increased angiotensin l)	[120]
	MO strains	Anti-inflammatory perspectives for colitis treatment	[121]

### 2.2. Water kefir acetic acid bacteria and their bioactive metabolites

#### 2.2.1. Acetobacter fabarum

The *Acetobacter fabarum* is one of the acetic acid bacteria (AAB) that has been identified as one of the dominant bacterial species in the water kefir microbial community [124], but has also been isolated from other fermented foods, with several metabolites being produced. Dietary acetoin, gluconic acid, and cellobiose produced by *A. fabarum* can exert anti-inflammatory and/or antioxidant activity, associated with their action on the intestinal microflora [125]–[127]. Also, mutated strains of this bacterium show improved ability to colonize the gut of organisms as part of the microflora [128]. Moreover, some experiments carried out in the *D. melanogaster* model confirm the significant activity of *A. fabarum* against aging and related neurodegenerative diseases [129],[130].

In addition, the *A. fabarum* DH1801 strain of Korean kefir, and its secreted metabolites have also exhibited probiotic antimicrobial activity against seven foodborne pathogens (*Bacillus cereus*, *Staphylococcus aureus*, *Listeria monocytogenes*, *Cronobacter sakazakii*, *Salmonella enteritidis*, enterotoxigenic *Escherichia coli*, and *Shigella flexneri*), as the culture filtrate from this strain's culture inhibited the growth of all seven pathogenic bacteria in a dose-dependent manner, which was superior to acetic acid solution of the same pH value. *A. fabarum* DH1801 strain forms a protective barrier during kefir fermentation against contamination by foodborne pathogens [131].

#### 2.2.2. Acetobacter orientalis

The AAB *Acetobacter orientalis* is one of the dominant species found in water kefir, where it acts as a fermentation stabilizer and contributes to the final product flavor [132]. Some strains of this bacterium have been detected in other fermented foods, such as Caspian Sea Yogurt [133]. Moreover, this bacterium is found in the gut microflora of *D. melanogaster*, being a key regulator of fly life span [134].

Apart from ethanol and acetic acid produced from *A. orientalis* when fermenting D-glucose, other metabolites produced, like the 2-keto-D-gluconic acid (2KGA) from D-glucose and lactobionic acid (LBA) from lactose, have shown several health-promoting effects. For example, 2KGA is used for the production of the food antioxidant erythorbic acid [135], while LBA possesses significant properties, including its antioxidant and probiotic activity, which make it applicable in the food, cosmetics, and pharmaceutical industries for the production of health-promoting products [136]. The presence of *A. orientalis* in water kefir fermentation was also associated with the presence of other bioactive metabolites too, such as several phenolic acids of different classes and molecular weights, as well as monoglycerides, isoleucine derivatives, and flavones [137].

Researchers have referred to the antioxidant activity of various fermented products produced by fermentation procedures in which *A. orientalis* participates. For example, cow's milk kefir showed significant antioxidant capacity at the ABTS radical scavenging activity assay but also strong antibacterial activity [123], while Caspian Sea Yogurt produced by milk inoculated with the Caspian Sea Yogurt bacterial community of *A. orientalis* and other strains showed a high phenolic content and significant antioxidant capacity as assessed by DPPH [138]. Moreover, onion vinegar derived by a two-stage fermentation process (initially with *Saccharomyces cerevisiae* to produce ethanol and then with *A. orientalis* to metabolize ethanol towards acetic acid) also showed antioxidant activity at both the DPPH and ABTS radical scavenging activity assays, with the vinegar product showing also a higher content of flavonoids and polyphenols but also a stronger antioxidant activity compared to various commercial vinegars [139].

#### 2.2.3. Acetobacter pasteurianus

The AAB *Acetobacter pasteurianus* is commonly present in plants and plant products and is widely used in the production of fermented foods, such as water kefir [137]. Many studies have reported the antioxidant and anti-inflammatory activity of vinegar produced by fermentation with *A. pasteurianus*. For example, vinegar fermented with *A. pasteurianus* under light-emitting diode (LED) conditions inhibited the production of IL-6 in LPS-stimulated RAW 264.7 cells [140]. Vinegar prepared Thai rice through a two-stage fermentation process, an alcoholic fermentation using the yeast *Saccharomyces cerevisiae* followed by acetic acid fermentation with *A. pasteurianus*, showed a high total phenolic content related to both strong antioxidant activities as assessed in ABTS and DPPH radicals, as well as to anti-cancer activity assessed in colon cancer cell lines [141]. Onion vinegar (OV) produced under the same fermentation process showed high total polyphenol and flavonoid content and *in vivo* antioxidant activity in *Caenorhabditis elegans* by enhancing the antioxidant enzymatic activities of glutathione peroxidase (GSH-Px), superoxide dismutase (SOD), and catalase (CAT) [142].

Strains of *A. pasteurianus* isolated from water kefir showed a high ability to produce EPS, which displayed strong antioxidant capacity and great anti-tyrosinase activity, with potential applications for the bacterium and its metabolites in the food and cosmetic industry for the production of relevant health-promoting products [143]. *A. pasteurianus* has also been found to alleviate the negative effects of alcohol consumption on cognitive function and liver health by modulating the gut microbiota-brain/liver axes in mice, as the presence of the bacterium resulted in improving alcohol-induced hippocampal damage, suppressing neuroinflammation, promoting hippocampal neuroprotein expression, and enhancing cognitive function. Concomitantly, *A. pasteurianus* ameliorated alcoholic liver injury by reducing serum lipid levels and oxidative stress, inhibiting the TLR4/MyD88/NF-κB pathway, and reducing TNF-α and IL-1β secretion. Also, treatment with *A. pasteurianus* enhanced the gut microbiota, inhibiting the growth of detrimental bacteria and promoting the recovery of beneficial bacteria [144].

#### 2.2.4. Acetobacter tropicalis

*Acetobacter tropicalis* is part of the AAB family of the water kefir microflora [51],[137]. For example, *A. tropicalis* has been isolated from kefir obtained from fermentation of brown sugar, purified molasses, and high-test molasses, which showed antihypertensive properties by inhibiting ACE, as well as antioxidant and antibacterial effects [31]. *A. tropicalis* is also an important source of B vitamins, including tetrahydrofolate (B9), riboflavin (B2), pyridoxine 5-phosphate (B6), biotin (B7), and was strongly correlated with the release of sulfur-containing metabolites [145], and the production of EPS bioactives [143]. *A. tropicalis* has also been found to possess probiotic benefits in *Drosophila melanogaster*, in which this bacterium has induced a reduction of lipid accumulation by adult *A. tropicalis*-colonized flies, which was linked with a parallel bacterial-mediated reduction in food glucose content. Thus, selective consumption of dietary constituents by microorganisms like *A. tropicalis* can alter the nutritional balance of food and, thereby, influence the nutritional status of the host [146].

**Table 2.** Bioactives and health promoting properties of water kefir AAB microorganisms.

AAB Microorganism	Bioactive compound	Health promoting effect	Ref
*Acetobacter fabarum*	Culture filtrate	Antimicrobial activity against foodborne pathogenic bacteria, with potential as a natural food preservative and probiotic agent	[131]
	Supernatant	Strong antibacterial activity, with potential probiotic properties	[147]
	Supernatant	Antifungal activity against important food originated mold species and antibacterial activity against one foodborne pathogen	[148]
	Fermentation of pasteurized whole cow milk with kefir grains containing, among other MO, *A. fabarum*-kefir and kefir fractions	Potent probiotic and therapeutic source against AD, as it improved the survival rate and neurodegeneration index of AD-like flies	[130]
*Acetobacter orientalis*	Fermentation of cow's milk with kefir grains containing, among other MO, *A. orientalis*-kefir beverage	Functional food product with significant antioxidant capacity and strong antibacterial activity	[123]
	Fermentation of milk with *Caspian Sea Yogurt* bacterial community *consisting of strains of A. orientalis* and *Lactococus lactis subsp. cremoris*-Caspian Sea Yogurt	Functional food product with increased total phenolic content and enhanced antioxidant activity	[138]
	Two-stage onion juice fermentation with *Saccharomyces*and then with *A. orientalis*-onion vinegar	Functional food product with increased total flavonoids and polyphenols content and enhanced antioxidant activity	[139]
*Acetobacter pasteurianus*	Total Phenolic Content	Antioxidant activity (ABTS and DPPH)	[141]
		Anti-cancer activity on colon cancer cell lines	[141]
	Vinegar fermented with MO	Anti-Inflammatory, inhibition of IL-6 production in LPS-stimulated macrophage cells	[140]
	Polyphenol and flavonoid content	High antioxidant activity	[142]
	EPS (Exopolysaccharide)	Strong antioxidant capacity and anti-tyrosinase activity	[143]
		Potential application in food and cosmetic industries	[143]
	The MO itself	Alleviation of alcohol-induced cognitive damage and liver injury	[144]
		Modulation of gut microbiota-brain/liver axes	[144]
		Suppression of neuro-inflammation and improvement of cognitive function	[144]
		Reduction of serum lipid levels and oxidative stress	[144]
		Anti-inflammatory, inhibition of TLR4/MyD88/NF-κB pathway and inflammatory cytokines	[144]
*Acetobacter tropicalis*	The MO itself	B vitamins source	[149]
	The MO itself	Angiotensin converting inhibition, antioxidant and antibacterial activity	[31]

### 2.3. Water kefir Bifidobacteria and their bioactive metabolites

#### 2.3.1. Bifidobacterium aquikefiri

*Bifidobacterium aquikefiri* was originally isolated from a domestic water kefir fermentation [150], and since then, it has also been found in a variety of water kefir fermentations, both on a household and industrial scale [151],[152]. The genome of *B. aquikefiri* possesses genes that give the bacterium the necessary characteristics to adapt to the water kefir ecosystem. *B. aquikefiri* strains belong to the genus *Bifidobacterium*, which is one of the most beneficial microorganisms with probiotic properties and associated health benefits, including the support of the immune system [150]. This bacterium can produce metabolites like lactic acid and mannitol with pleiotropic effects on the inflammatory process [153] and suggested anti-inflammatory and antioxidant potential [154]–[156].

*B. aquikefiri* possesses genes encoding for enzymes that are involved in the production of several amino acids, which support the ability of this bacterium to fully synthesize such metabolites, including L-alanine, L-glutamine, L-proline, and L-glycine [157], which have been reported for their significant antioxidant activity [158]–[161]. *B. aquikefiri* can also produce pyridoxal 5-phosphate, the active form of vitamin B6, from glutamic acid and glyceraldehyde 3-phosphate [41]. Moreover, researchers investigating the microflora and constituents of a water kefir sample found that the presence of *B. aquikefiri* was strongly and positively correlated with the levels of 2-phenylethyl acetate, the alcohols 2-phenylethanol, 2-methylbutanol, and 3-methylbutanol, as well as succinic acid [152], which has exhibited good antioxidant and antibacterial properties [162].

In contrast to other *Bifidobacteria* [163], *B. aquikefiri* harbors genes encoding for SOD that catalyzes the dismutation of O^2−^ to O_2_ and H_2_O_2_, as well as for GSH-Px, which contributes to the maintenance of redox homeostasis of this strain under stress response using reduced glutathione to quench oxidative radicals. The presence of both these enzymes might contribute to antioxidant health benefits for this MO, since SOD constitutes a very important antioxidant defense against oxidative stress in the body and is a good therapeutic agent against reactive oxygen species (ROS)-mediated diseases [164], while GSH-Px activity is a primary antioxidant defense system that plays a key and fundamental role in the overall defense mechanisms and strategies in biological systems and has been implicated in the prevention of the development of many common and complex diseases, including cancer and cardiovascular disease [165]. However, more targeted research is needed to fully elucidate the health-related potential of the presence of the genes encoding for these two important antioxidant enzymes in *B. aquikefiri*.

#### 2.3.2. Bifidobacterium crudilactis

*Bifidobacterium crudilactis* has been detected in water kefir [166], but also in many other food products. Some studies highlight the ability of this bacterium to grow at 12 °C during the production of raw milk cheeses, as well as to tolerate the high temperatures applied during heat treatment of milk [167]. These properties of the bacterium enable it to be used in new foods and food supplements, and also in products whose preparation requires some heat treatment. In addition, the demonstrated ability of *B. crudilactis* to survive in the presence of oxygen, as well as at low pH and temperature, makes it a candidate strain with probiotic potential [168].

When *B. crudilactis* grew in a culture medium containing 3′-sialyl-lactose as a major carbon source, a culture supernatant was obtained with a high content of short-chain fatty acids (SCFAs), mainly propionic acid, with a significant amount of these SCFAs being derived from the unfermented culture medium [169], while such SCFA metabolites of the gut microbiota can regulate the inflammatory response of the host, contributing to its homeostasis. More specifically, these bioactive products can mitigate inflammation by regulating the production of cytokines by immune cells [170], and several *in vivo* and *in vitro* studies have highlighted the significant anti-inflammatory activity of SCFAs [171],[172].

The available experimental data concerning the bioactivity of *B. aquikefiri* and *B. crudilactis* are limited and mainly focus on the probiotic activity of *B. crudilactis* [169],[173]. Nevertheless, the antioxidant activity of water kefir samples obtained from the fermentation of water kefir grains containing, among other microorganisms, the above two bacteria has recently been reported [174]. Kefir samples were prepared using sugar or fig as fermentation medium. Both kefirs showed strong antioxidant capacity, which was determined by the TEAC (Trolox Equivalent Antioxidant Capacity) and ORAC (Oxygen Radical Absorbance Capacity) methods. Interestingly, the fig-based water kefirs showed higher antioxidant activity than those prepared with sugar.

#### 2.3.3. Bifidobacterium psychraerophilum

*Bifidobacterium psychraerophilum* has been found in various fermented food products, including water kefir [175], but also as a probiotic in the microbiota of the gut and fecal microflora of flies. *B. psychraerophilum* produces mainly acetic acid, formic acid, as well as lactic acid, while it possesses a complete mevalonate pathway for the biosynthesis of isoprenoids [149]. Moreover, some fatty acids, including the C18:1, C16:0, and C18:0 fatty acids, as well as several glycolipids and specific phospholipids, are present in cells of *Bifidobacteria*, with strains of *B. psychraerophilum* being capable producers of conjugated linoleic acids (CLAs) [176], for which a complex effect on several inflammatory pathways has been reported [177]. The anti-inflammatory effects of some CLAs have been proposed to be mediated by the inhibition of the expression of pro-inflammatory cytokines and chemokines, such as the Intercellular Adhesion Molecule 1 (ICAM-1) through the blocking of NF-κB transcriptional regulation and the attenuation of MAPK signaling pathways [178].

### 2.4. Water kefir bacteria of the *Zymomonas* genus and their bioactive metabolites

The most abundant-dominant representative specie of this genus in water kefir is the ethanol-producing bacterium *Zymomonas mobilis* [26]. This important bacteria also occurs naturally in various types of fermentation products [179]. *Z. mobilis* is a Gram-negative and facultative anaerobic alpha-proteobacterium reported to produce biofuel and bioproducts andis useful for applications in the food industry and healthcare [180],[181]. Some studies have revealed that, besides the high ethanol productivity, fermentation in *Z. mobilis* also leads to a high yield of levan, a fructose polymer that is synthesized only when sucrose or another suitable sugar, such as raffinose, is present in the culture medium of *Z. mobilis* [182]. In contrast, levan production has not been reported in cultures grown on glucose, fructose, or a mixture of these. Its formation is attributed to levan sucrase, one of the enzymes that act on sucrose [182]. Levan, produced by *Z. mobilis*, has been proposed as a cosmetic ingredient [181], while the anti-inflammatory potential of levan against skin inflammation induced by sodium lauryl sulfate (SLS) has been reported, as a reduction of the levels of pro-inflammatory IL-1a was observed in a three-dimensional (3D) artificial skin treated with levan (0.01 and 0.05 mg/mL) in comparison to the skin sample treated only with SLS.

*Z. mobilis* produce several other metabolites with a variety of applications, among which are various enzymes, used in diagnostic analysis and research [183], as well as R-phenyl acetyl carbinol (PAC), used by the pharmaceutical industry as a precursor compound for the production of ephedrine and pseudoephedrine [180]. Moreover, several genes have been introduced into the *Z. mobilis* genome to produce a wider range of metabolites that can also be used as high-value products, such as L-alanine [184], D-lactate [185], and beta-carotene [186], which has shown antioxidant and anti-inflammatory potential [187],[188].

Interestingly, several lipid bioactive metabolites have also been observed to be present in *Z. mobilis*, and thus its lipid composition has been studied by several researchers. Cells of this bacterium consist of 6.3% extractable lipids and 1.5% bound lipids, with 96.8% of the extractable lipids being polar lipids, while the remaining 3.2% are neutral lipids [189]. Regarding the fatty acids of the *Z. mobilis* membrane, vaccenic acid (*trans* 18:1n7) is the most abundant, followed by myristic acid (14:0), palmitic acid (16:0), palmitoleic acid (16:1n7), and stearic acid (18:0), while traces of lauric acid (12:0) and myristoleic acid (14:1) were alsopresent [190],[191]. Several of these fatty acids, including myristic acid [192], palmitoleic acid [193],[194], stearic acid [195],[196], and lauric acid [197],[198] have been reported to possess antioxidant and anti-inflammatory properties.

By utilizing a one-step high-performance liquid chromatography (HPLC) analysis, the total unfractionated lipid extract of *Z. mobilis* was able to be separated into lipid fractions of each lipid class present in this bacterium, while both the total lipid extract of this bacterium and all its fractionated lipid subclasses collected by this analysis were further assessed for their potential anti-inflammatory and antithrombotic potential in bioassays performed on washed rabbit platelets [199]. It was observed that the most bioactive lipid classes of *Z. mobilis* were its polar lipid bioactives and especially the fractions of glycolipids and the more amphiphilic phenolics or phenolipid molecules, which potently inhibited the thrombo-inflammatory action of the potent inflammatory mediator, platelet-activating factor (PAF), and its associated induction of platelet activation and aggregation. In the applications front, the molecular identification of PAF inhibitors in this bacterium is of considerable pharmacological interest, as has been the case with other well-established bioactive molecules of similar function that are derived from natural sources, including bio-functional microorganisms, which are used as standard drugs, food supplements, or as forefront standard PAF-antagonists in research studies [20].

Other studies have also shown potential anti-inflammatory health benefits for this bacterium [200]. For example, the combination of prophylactic and therapeutic treatment (pre-treatment and/or post-treatment) with *Z. mobilis* UFPEDA 202 (109 CFU/mL) cultures on polymicrobial sepsis, induced by cecal ligation and puncture (CLP), in a sepsis model of male mice, increased the survival of the mice by 50% at 96 h after sepsis induction. Indeed, an anti-inflammatory reduction in the levels of TNF-α and myeloperoxidase (MPO) in lung tissue was observed, as well as a decrease in the number of viable bacteria in the peritoneal fluid. Furthermore, there was an increase in neutrophil migration and in the levels of the anti-inflammatory cytokine IL-10, while histopathological analysis revealed a reduction in acute lung injury. In addition, mice pretreated with *Z. mobilis* showed a significant reduction (by 24%) in the number of apoptotic cells in the spleen.

All the above studies demonstrate the anti-inflammatory activity of *Z. mobilis*, making it a potential platform for producing a multitude of biofunctional products (functional foods, cosmetics, etc.) capable of combating oxidative stress and inflammation. Therefore, further study of the antioxidant, anti-inflammatory, and antithrombotic activity of *Z. mobilis* is deemed necessary.

Fermented foods play a vital role in people's diets worldwide, as their consumption has been found to promote health [201], while the beneficial properties of such fermented products are due to the presence of bioactive microorganisms or substances produced by them, with the bacterium *Z. mobilis* being identified in a variety of fermented products and especially in alcoholic beverages [201]–[204]. Several studies have also referred to the antioxidant-beneficial effects of *Z. mobilis* fermentation products. For example, *Z. mobilis* was found to be one of the dominant species in the bacterial community of the Tej beverage, an Ethiopian wine made from honey, which showed significant antioxidant activity in both the DPPH and ABTS radical scanning assays [205]. Kombucha, a traditional fermented beverage in which *Z. mobilis* has been detected, showed antioxidant potential too, which was determined using the oxygen radical absorbance capacity (ORAC) [206]. Finally, a lacto-vinegar functional product, which was produced by fermentation with *Acetobacter pasteurianus* of an alcoholic beverage that was initially produced by fermentation with *Z. mobilis* of a whey solution saccharified with Koji (prepared from rapeseed meal or wheat bran), showed high nutritional and functional properties, including stronger antioxidant activities than the ordinary commercial vinegar [207].

**Table 3.** Bioactives and health promoting properties of other water kefir microorganisms from the *Bifidobacteria* and *Zymomonas* genus.

Microorganism	Bioactive compound	Health promoting effect	Ref
*Bifidobacterium aquikefiri* and *Bifidobacterium crudilactis*	Fermentation of sugar-based or fig-based medium with water kefir grains containing, among other MO, *B. aquikefiri* and *B. crudilactis*-water kefir samples	Functional food products with strong antioxidant activity	[174]
*Bifidobacterium crudilactis*	*B. crudilactis* in co-culture with 3′-sialyllactose	Potent combination for a favorable to the gut health of young children formula, as it tends to a bifidogenic effect on toddler microbiota	[169]
*Zymomonas mobilis*	Levan produced by *Z. mobilis*	Anti-inflammatory effect against inflammatory reactions to skin irritants (reducing the secreted amount of pro-inflammatory mediator IL-1a induced by SLS in bio-artificial skin), with potential applicability as a cosmeceutical agent	[181]
	Lipids extracted from *Z. mobilis*	Potent inhibitory activity against PAF thrombo-inflammatory action, with health promoting prospects in pharmaceutical industry applications against inflammation and thrombosis	[199]
	*Z. mobilis* cultures	Protective effect against polymicrobial sepsis induced by CLP sepsis model in mice, by modulating the inflammatory response (increasing the levels of IL-1a and reducing the levels of TNF-a and MPO in lung tissue), alleviating bacterial burden and suppressing splenocyte apoptosis	[200]
	Tej beverage containing as a dominant specie *Z. mobilis*	Functional food product with strong antioxidant activity	[205]
	Kombucha beverage containing as a dominant specie *Z. mobilis*	Functional food product with strong antioxidant activity	[206]
	Lacto-vinegar produced by fermentation with *Acetobacter pasterianus* of an alcoholic beverage obtained by a *Z. mobilis* based fermentation of a whey solution saccharified with Koji	Food product with high nutritional and functional properties, including strong antioxidant activity	[207]

### 2.5. Water kefir fungi and yeasts and their bioactive metabolites

#### 2.5.1. Dekkera anomala

*Dekkera anomala* is a member of the *Brettanomyces* genus yeast, commonly found in water kefir [26], as well as in other fermented products. The presence of such yeasts in kefir contributes to B vitamin synthesis, which in turn assists microbial growth in the kefir. It has not been proved that *D. anomala* has a primary role in increasing the antioxidant activity of water kefir, but it has been found in products that have shown antioxidant potential [92],[208]. However, this yeast is used in the market as a probiotic and can also be utilized in functional foods and other applications.

For example, *D. anomala* has been referred in patents for fermentation procedures producing active metabolites for specific cosmetic products and applications [209]. In addition, ethylphenols, which have been identified as unfavorable off-ﬂavors, often called “brett” ﬂavors, produced by *D. anomala* during fermentative production of wines and other beverages, are instead desired in particular products. More specifically, ethyl phenols are used in the food industry as flavoring agents in foods and beverages due to their strong aroma, while 4-ethylphenolis used in cosmetic formulations as an important cosmetic ingredient that is commonly used as a fragrance ingredient and flavoring agent. Additionally, 4-ethylphenol seems to act as an effective depigmenting agent [210], with reported hypopigmentary effects by attenuated mRNA and protein expression of tyrosinase-related protein (TRP)-2, as well as antioxidative activities, by inhibiting lipid peroxidation, which can help to protect the skin from damage caused by free radicals and hyperpigmentation. While it is generally considered safe for use and has been found to have some potential health benefits, such as antioxidant and anti-inflammatory properties, this *D. anomala* metabolite can also negatively affect human health if consumed and/or used in large quantities, such as skin irritation.

#### 2.5.2. Dekkera bruxellensis

*Dekkera bruxellensis* is a yeast widely studied in several beverages, including water kefir [211] and kombucha [212]. Notably, *D. Bruxellensis* has been closely compared to *Sacharmomyces Cerevisiae*, for example, in their effect on phenolic constituents [213],[214]. *Dekkera* is almost unique among other yeasts because of its ability to convert hydroxycinnamic acids–antimicrobial non-volatile compounds present in grape must–into ethyl derivatives [215]. In the various products that this yeast was studied in, it showed antioxidant potential [212]. Moreover, apart from *D. anomala*, *D. bruxellensis* also contributes to the production of the off-ﬂavors, ethyl phenols, in wines and other beverages, which as aforementioned, are used in cosmetic applications, especially as effective skin depigmenting agents [210] and antioxidants for skin care. In another study, *D. bruxellensis* was used as a starter yeast to ferment and mix sweetened black tea and wheatgrass juice, which demonstrated a higher, more stable antioxidant activity closely related to the increase of phenolic compounds, flavonoids, and anthocyanidin content [216]. Moreover, researchers suggested that the effects of the microorganisms and their metabolites in kombucha may be due to the secondary fermentation additives. In detail, the DPPH assay showed that primary fermented kombucha and dried ginseng kombucha had the highest antioxidant activity, and the NO assay showed that kombucha fermented with grapefruit and ginseng had excellent anti-inflammatory activity [217].

#### 2.5.3. Hanseniaspora valbyensis

*Hanseniaspora valbyensis*is a yeast that has also been found to be present in water kefir [211] and kombucha [218]. Bioactive metabolites associated with *H. valbyensis* are ethyl acetate, 2-phenyl acetate, and phenylethyl acetate, among others [219], for which antibacterial and antioxidant activity has been reported [220]. Interestingly, *H. valbyensis* isolated from kombucha showed strong antioxidant potential in comparison to all the other isolated MO from this source [208].

#### 2.5.4. Lachancea fermentati

*Lachancea fermentati* is a fungi detected in water kefir [38], kombucha cultures [221], and other fermented sources. *L. fermantati* has shown resistance in several drug tests [222] and seems to have future potential as a probiotic.

#### 2.5.5. Saccharomyces cerevisiae

*Saccharomyces cerevisiae* is one of the most utilized yeasts for producing functional fermented products and especially for the production of several ethanolic beverages, including wine, apple cider, and beer [15],[18],[20],[223]. It is a probiotic fungi with high tolerance in bile salts, high NaCl concentrations, simulated gastric juice, and the intestinal environment, as well as in drugs like tetracycline, ampicillin, penicillin, gentamycin, polymyxin B and nalidixic acid, and inhibitory effects against α-amylase, trypsine, and lysozyme [224]. *S. cerevisiae* is generally classified as aprobiotic yeast with beneficial immunomodulatory properties. However, intestinal microbial changes such as a decrease *S. cerevisiae* is a common feature of chronic inflammatory diseases like psoriasis and inflammatory bowel disease (IBD), suggesting the presence of a gut-skin axis. Dimethylfumarate (DMF) therapy showed that it can restore the depletion of *S. cerevisiae* in psoriasis patients, as the DMF use raisedfecal *S. cerevisiae* levels [225], while anti-*Saccharomyces cerevisiae* antibodies were not elevated in psoriasis. Another important use of *S. cerevisiae* as probiotics in skin and gut homeostasis is its contribution in inflammation reduction, giving great perspectives against vaginal candidiasis too [226]. Moreover, *S. cerevisiae* was used to biosynthesize seleno proteins from inorganic selenium, with these protein products exhibiting enhanced antioxidant activities, according to scavenging activity tests [227].

Since it's a lactic acid producer, it has been engineered for rosmarinic acid and angelic acid production, which are very functional metabolites, with rosmarinic acid exhibiting antioxidant, anti–inflammatory, antibacterial, antiallergic, and antiviral properties, while anticancer and anti-inflammatory activities have been reported for angelic acid too [228],[229].

*S. cerevisiae* strains exhibited *in vivo* reduction of pro-inflammatory cytokines IL-1β, IL-6 and TNF-α and promoted the expression of the anti-inflammatory cytokine IL-10 in colitis mice [230]. Similarly, in DSS-induced colitis mice, *S. cerevisiae* strain QHNLD8L1 contributed in IL-1β reduction and IL-10 increase, with further inflammation response regulation [231]. *S. cerevisiae* strain IFST 062013, isolated from fruit, showed probiotic activity through assimilation of cholesterol and anti-inflammatory effects by producing several important compounds with immunomodulatory effects in T-lymphocyte proliferative response, suggesting also an antitumor potential for *S. cerevisiae* [224].

Finally, it has recently been found that *S. cerevisiae* is one among several other yeasts used for the production of fermented foods and beverages, which contain highly bioactive polar lipids and phenolics with strong anti-inflammatory and anti-thrombotic properties against the thrombo-inflammatory mediators, PAF and thrombin, as well as against the well-established platelet agonists, collagen, and ADP [15]–[18],[232]. It seems that the anti-inflammatory and anti-thrombotic anti-PAF properties of these yeast-derived polar lipid bioactives can subsequently reduce the risk for PAF-associated inflammatory chronic disorders, such as atherosclerosis, CVD, and cancer [6],[15],[16],[18], suggesting that these metabolites of *S. cerevisiae* are also promising candidates as ingredients for producing novel functional products with anti-inflammatory health-promoting effects.

#### 2.5.6. Zygosaccharomyces lentus

*Zygosaccharomyces lentus* is also one of the yeasts present in water kefir and in other fermented beverages [233], including symbiotic cultures of bacteria and yeast (SCOBY), in kombucha tea production [234]. *Z. lentus* possesses an interesting tolerance against food additives [235], as well as tolerance to high NaCl concentration solutions and perspectives for flavor properties in beverages and other useful applications [217]. Some innovative applications of *Z. lentus* present in kombucha fluorescent powder have also been evaluated for their use in the extraction of rare earth elements (REE), proven to be very useful and sustainable [236].

#### 2.5.7. Zygotorulaspora florentina

*Zygotorulaspora florentina* is a fungi isolated from both dairy and water kefir [237]. Its biochemical activity for lipid accumulation and lipid production in oleaginous kefir yeast with several applications has been evaluated [238]. After examination of different fermentation methods, using *Z. florentina* and *S. cerevisiae* strains, their interactions were also evaluated. Pure *Z. florentina* strains were characterized by increased available amino nitrogen in contrast with mixed fermentations where the cell concentrations were undetectable but mannoproteins, 2-phenylethanol, and other compounds were present, revealing potential perspectives in wine production. It seems to have a potential role in beer, wine production processes, and dough fermentation [239]. Additionally, mannoproteins derived from cells of other yeasts have displayed significant bioactivity, including antioxidant and anticancer activity [240].

**Table 4.** Health promoting properties of water kefir fungi and yeasts.

Microorganism	Bioactive compound	Health promoting effect	Ref
*Dekkera Bruxellensis*	products of MO	Antioxidant activity potential	[215]
	Ethylphenols	Skin depigmenting agent and antioxidant	[210]
	Phenolic compounds	Increased antioxidant activity and stability	[216]
	Flavonoids	Enhanced antioxidant activity	[216]
	Anthocyanidins	Antioxidant activity	[216]
	Fermentation of kombucha by MO, with ginseng additive	High antioxidant and anti-inflammatory activity	[217]
	Fermentation of kombucha by MO, with grapefruit additive	Anti-inflammatory activity	[217]
*Hanseniaspora valbyensis*	The MO itself	Antioxidant activity	[208]
*Saccharomyces cerevisiae*	DMF cocultivation with *S. cerevisiae*	Anti-inflammatory and immunomodulatory effects (β-glucans production)	[225]
	Seleno proteins biosynthesis	Antioxidant activity	[227]
	Rosmanirid acid production	Antioxidant, anti-inflammatory, antibacterial, antiallergic and antiviral activity	[228]
	Angelic acid production	Anticancer and anti-inflammatory activity	[229]
	The MO itself in probiotics	Inflammation suppression, potential use against vaginal candidiasis	[226]
	*In vivo* strains	Colitis prevention	[230]
		Colitis inflammation reduction	[231]
		Probiotic and anti-inflammatory	[224]
		Anti-inflammatory and antithrombotic	[15]–[18],[232]

## 3. Antioxidant, anti-inflammatory, and antithrombotic health promoting properties of water kefir and its fermented bio-functional products

Several beneficial properties and health-promoting effects of water kefir, including its antioxidant, anti-inflammatory, and antithrombotic activities, have been attributed to its symbiotic microorganisms and the metabolites they produce, which was discussed in detail individually for each MO present in water kefir in section 2.

In any case, it should be considered that, although live microorganisms present in the fermented drink can exert the positive effects attributed to water kefir, it seems that the biological activities of water kefir are derived by their combination, interactions, and the co-presence of their metabolic products.

Thus, here we present the reported health promoting properties of water kefir as a whole symbiotic microbiota in classic water kefir-based functional beverages as well as in several other fermented products with anti-inflammatory and antioxidant bio-functionalities and associated health benefits.

### 3.1. Antioxidant and anti-inflammatory effects of the water kefir beverage

The antioxidant activity of water kefir was first reported in 2013 when a sample of fresh water kefir showed a high ability of scavenging DPPH radicals and inhibiting ascorbate autoxidation. This was attributed to the lactic and acetic acid bacteria and yeasts found in kefir, their intracellular and extracellular metabolites, and to their cell lysis products [241]. Several recent *in vitro* studies have also demonstrated the antioxidant capacity of this beverage. In one of them, increasing the fermentation time induced a significant enhancement of the antioxidant activity of the produced kefir water, which was determined by DPPH, FRAP, cupric reducing antioxidant capacity (CUPRAC), and potassium ferricyanide reducing power (PFRAP) assays [242]. In another similar study, the application of the DPPH method revealed the high antioxidant capacity of water kefir samples produced from different fruit juices [243]. Moreover, kefir beverages obtained from the fermentation of water containing mandarin and persimmon showed high antioxidant activity too. In fact, although both of these beverages showed similar DPPH values, the mandarin-based kefir had a higher trolox equivalent antioxidant capacity (TEAC) value [244]. In all these studies, the strong antioxidant capacity of the water kefir beverage was associated with its high content of phenolic components, which was determined by the Folin-Ciocalteau colorimetric method.

The antioxidant activity of this drink has also been confirmed by many *in vivo* studies. In one of them, the administration of water kefir to mice resulted in an increase in superoxide dismutase (SOD) activity and plasma iron reduction capacity (FRAP), as well as a reduction in nitric oxide levels, mainly in brain and kidney samples. The antioxidant activity of kefir was attributed to the flavonoids and phenolic acid derivatives contained in it, the presence of which was detected by analytical methods such as ultra-high-performance liquid chromatography (UHPLC). These results, along with the toxicological screening performed, highlighted water kefir as a safe source of antioxidants for daily consumption [51]. In an additional *in vivo* experimental study in mice, water kefir showed gastroprotective and antioxidant capacity, improving protein oxidation and antioxidant enzyme activity [245].

The anti-inflammatory effect of water kefir has been established, both *in vivo*, with granuloma and leg edema tests in rats [246]–[248], and *in vitro*, with the red blood cell membrane stabilization method [248]. In all *in vivo* studies, treatment with water kefir led to suppression of granulomatous tissue formation and edema. This anti-inflammatory activity of the beverage was linked to its ability to stabilize the red blood cell membrane [249]. In a different study, the potential benefits of water kefir against cancer were suggested by its observed antimetastatic, and antiangiogenic effects, since this fermented beverage inhibited the tumor proliferation *in vitro* and *in vivo* mainly by promoting cancer cell apoptosis, immunomodulation by stimulating helper and cytotoxic T cells, and anti-inflammatory, antimetastatic, and anti-angiogenesis effects [30].

Many researchers have also highlighted the role of water kefir in promoting gut health. In one of them, which was performed using an artificial colon setup, the effect of pasteurized and unpasteurized water kefir products on the intestinal microbiota, epithelial barrier function, and immunomodulation was investigated. The results revealed that kefir water treatment resulted in a positive regulation of the colonic microenvironment, increasing the production of SCFAs and simultaneously reducing detrimental fermentation proteolytic compounds. Water kefir also induced an increase in the abundance of *Bifidobacterium*, as well as enhancing the epithelial barrier. Interestingly, pasteurized kefir products showed enhanced benefits, improving inflammation-induced intestinal epithelial barrier disruption and increasing IL-10 and IL-1β [250], which may indicate that water kefir metabolites released in the pasteurized product are the responsible ones for these health benefits.

In addition, specific *Lactobacillus* strains isolated from water kefir showed antioxidant activity and potential probiotic properties, including the ability to survive at low pH, bile salt tolerance, and the ability to adhere to intestinal cells [251]. In a study conducted *in vivo* in a mouse model, the prophylactic and therapeutic effects of water kefir on irritable bowel syndrome (IBS) were demonstrated. More specifically, water kefir induced a reduction in the expression of the pro-inflammatory cytokines TNF-α and NF-κB, and thus it's associated expression of inflammatory genes in this animal model [252].

Several studies have revealed the ability of kefir to protect against neurodegeneration by acting as an antioxidant and/or anti-inflammatory agent [253],[254]. Most refer to the bioactivity of kefir produced from fermented milk. However, a recent study established the neuroprotective effect of water kefir in H_2_O_2_-induced human neuroblastoma SH-SY_5_Y cells, facilitated by the antioxidant and anti-apoptotic activities of the beverage, which were also demonstrated. The results of the research suggested that the antioxidant, anti-apoptotic, and neuroprotective effects of water kefir were mediated through up-regulation of SOD and CAT antioxidant enzymes, as well as through modification of apoptotic genes [255].

Moreover, researchers investigated the impact of water kefir on body weight, blood glucose levels, and lipid profiles in normal and streptozotocin-induced diabetic Wistar rats in order to evaluate if water kefir can potentially be used for diabetes mellitus sufferers to control glucose and lipid levels due to proposed antihyperglycemic and antikyperilipidemic activities [256]. Water kefir prevented the weight loss often seen in diabetic rats by increasing body weight in both normal and diabetic rats with a 10% concentration. In addition, water kefir significantly reduced blood glucose levels in diabetic rats, with reductions of up to 71% compared to the diabetic control group, potentially through improved insulin sensitivity or altered glucose metabolism pathways. Furthermore, kefir demonstrated antihyperlipidemic properties by decreasing lipid profiles (total cholesterol, triglycerides, LDL, and VLDL) and improving HDL levels, which may help reduce cardiovascular risk associated with diabetes. These effects are suggested to be mediated by regulation of lipid metabolism and antioxidant capabilities [256]. Similarly, in another study after an *in vivo* examination of biochemical, physiological, and nutritional parameters, an improvement in the lipid profile of Wistar rats that consumed water kefir made with brown sugar has also been reported, suggesting a potential of water kefir against cardiovascular diseases [257].

Additional research evaluated the hepatoprotective activity of water kefir, particularly focusing on its impact on liver enzymes and inflammatory markers in a rat model of CCl4-induced liver injury. The results showed that treatment with water kefir significantly reduced serum ALT and AST levels. On top of that, water kefir treatment led to decreased levels of the pro-inflammatory cytokine TNF-α and TGF-β levels. Histological analysis in rats treated with water kefir revealed beneficial properties against liver necrosis. Furthermore, molecular docking studies highlighted key metabolites in water kefir that interacted with NF-κB and nrf2 Keap1 proteins, which are essential for inflammation and oxidative stress regulation. Also, fumaric acid and 2-phenylacetaldehyde showed strong interactions with NF-κB and nrf2 Keap1, which indicates potential hepatoprotective properties of water kefir. Notably, these interactions were facilitated by hydrogen bonding and other molecular interactions, suggesting a mechanism by which kefir components may modulate inflammation and oxidative stress pathways in the liver. Waterkefir demonstrated hepatoprotective effects in the liver-injury rat model, characterized by reductions in liver enzyme levels, inflammation markers, and histological improvements [258].

Moreover, the microbial community of water kefir exhibited a protective role in DSS-induced colitis in mice [259], by restoring the abnormal expression of pro-inflammatory and anti-inflammatory cytokines (i.e., IL-1β, IL-6, TNF-α, COX-2, iNOS, and IL-10) and the inactivated Toll-like receptor-4 (TLR4)-myeloid differentiation protein primary response 88 (MyD88)-NF-κB pathway. Furthermore, several studies have demonstrated the inhibitory effect of polysaccharide extracts isolated from water kefir grains on induced inflammation and subsequent edema in rat legs [74],[247],[260]. In addition, researchers identified certain extracellular enzymes from the microorganisms of water kefir as potential inhibitors of Nrf2, a major mediator of inflammation and oxidative stress [261].

Kefir and its insoluble polysaccharide, kefiran, have also shown strong antimicrobial and cicatrizing activities against several bacterial species and *Candida albicans*, while cicatrizing experiments using a 70% kefir gel had a protective effect on skin connective tissue and 7 day treatment enhanced wound healing compared with 5 mg/kg of neomycin–clostebol emulsion on Wistar rats with induced skin lesions and *Staphylococcus aureus* inoculation [262].

The ability to prevent gastric lesions was evidenced by Brasil et al. (2019) [263], where the pretreatment with water kefir for 14 days promoted a great antioxidant activity that protected the gastric epithelium.

**Table 5.** Antioxidant and anti-inflammatory health-promoting effects of water kefir and its beverages.

Water kefir usage	Health promoting property	Ref
Fresh water kefir	Antioxidant activity	[241]
water kefir produced increasing fermentation time	Antioxidant activity	[242]
Water kefir from fruit juices	Antioxidant activity	[243]
Water kefir beverages with mandarin and persimmon	Antioxidant activity with increased TEAC value	[244]
Water kefir in mice	SOD increase reduction of nitric oxide levels in brain and kidney, safe antioxidants evaluation	[51]
Water kefir in mice	Gastroprotective and antioxidant activity	[245]
Water kefir in vivo and in vitro	Anti-inflammatory activity	[74],[124],[246],[247],[249]
Water kefir in mice with breast cancer	Antimetastatic and antiangiogenic activity in 4T1 cancer cells	[30]
Water kefir pasteurized products	Inflammation induced intestinal epithelial barrier disruption, IL-10 and IL-1β increase	[250]
Water kefir MO strains	Antioxidant and probiotic activity	[251]
Water kefir in mice with IBS	Suppression of pro inflammatory cytokines TNF-α and NF-ΚB and inflammatory genes	[252]
Water kefir in H2O2 induced human neuroblastoma SH-SY5Y cells	Antioxidant, anti-apoptotic and neuroprotective effect	[255]
Water kefir	Neurodegeneration protection as antioxidant and anti-inflammatory	[253],[254].
Water kefir in streptozotocin induced diabetic rats	Body weigh increase, blood glucose reduction, antihyperlipidemic activity and potential cardiovascular protection	[256]
Water kefir in CCL4-induced animal model	Anti-inflammatory activity with liver damage improvement, suppression of AST, ALT, TNF-α, TGF-β serum levels and interaction with target proteins of NF-κB and Nrf2 pathways	[258]
Water kefir Microflora	Protective role in DSS-induced colitis in mice, modulation of cytokine expression, and TLR4-MyD88-NF-κB pathway	[259]
Water kefir Microflora derived Polysaccharide Extracts	Inhibitory effect on induced inflammation and edema in rat legs	[74],[247],[260]
Water kefir Microflora derived Extracellular Enzymes	Potential inhibitors of Nrf2, a mediator of inflammation and oxidative stress	[261]
Water kefir gel	Protective effect on skin connective tissue and wound healing activity	[262]

### 3.2. Antioxidant and anti-inflammatory health-promoting effects of other functional products produced by fermentations based on water kefir

The beneficial health-promoting effects of several other fermented bio-functional products that are produced by fermentations based on water kefir grains and using other substrates than those used in classic water kefir beverages, have also been reported [37],[74],[264]. For example, a tomato seed extract obtained from tomato seeds and subsequently fermented using water kefir grains exhibited strong antioxidant activities [265]. More specifically, the fermentation of the extract resulted in the enhancement of its antioxidant activity, which was evaluated by DPPH and ABTS assays. In a different study, water kefir microflora was used in the fermentation of soy whey [266]. The product that was obtained constituted a bioactive beverage with many beneficial effects, including antioxidant activity. A high antioxidant capacity has also been demonstrated using different substrates for fermentation with water kefir grains such as soybean hydrolyzed extract, colostrum, and honey [37], cornelian cherry, hawthorn, red plum, rosehip, and pomegranate juices [267]. In these studies, the strong antioxidant activity of the fermentation products was evaluated by the DPPH method and correlated with their detected high content of bioactive phenolic compounds. Moreover, a related study evaluated the shelf life of non-alcoholic beverages fermented with water kefir grains using red pitaya or red pitaya and apple pulp as a substrate [268]. Both beverages showed high antioxidant activity. However, the addition of apple pulp to the beverage fermented with red pitaya alone enhanced its antioxidant effects due to the high phenolic content of apple pomace. In another study, fermentation of pomegranate juice with a *Lactobacillus paracasei* SP3 strain isolated from water kefir grains enhanced its antioxidant activity [93]. Furthermore, the total phenolic content of the beverage increased. This fact was attributed to the transformation of the phenolic compounds contained in the juice, due to some enzymes involved in the metabolic activities of *L. paracasei* strains.

The high total phenolic content of water kefir fermentation products has also been reported by two very recent studies [269],[270], studying the evaluation of fermented quinoa protein concentrates and fermented demineralized whey. In addition, a recent *in vitro* study demonstrated a significant increase in the antioxidant capacity of red beetroot juice when it was fermented with water kefir grains [271]. Specifically, the obtained fermented juice showed an increased ability to scavenge free radicals of OH, O2−, ABTS·+, and DPPH·.

With respect to the anti-inflammatory properties of water kefir fermentation products, these have been studied both *in vitro* and *in vivo*. For example, in a related *in vivo*, study the anti-inflammatory effects of beer fermented by water kefir were investigated, compared to those found separately in kefir-souring molasses and craft beer, using carrageenan-induced edema in rat paws as a challenge model [264]. The results revealed a significant reduction in hind paw edema for rats treated with kefir beer as well as control beer modified with aqueous kefiran, whereas treatment with plain control beer did not induce an effective inhibition. Thus, it was concluded that the anti-inflammatory activity of beer was enhanced due to its fermentation by water kefir.

Evaluation of antioxidant activity and renal cell damage protection has also been evaluated using orange water kefir in hyperlipidemic rats [272]. Likewise, using soy whey to transform water kefir consortium into a bioactive beverage, resulted in interesting health-promoting effects, in addition to those induced by soy whey itself, such as an enhancement of its antihypertensive ACE inhibitory effect [266]. Moreover, hepatoprotective activity for fermented water kefir has also been reported against acetaminophen-induced liver toxicity *in vivo*, as it led to the suppression of both AST and ALT hepatic enzymes in normal, promoting thus liver health homeostasis [273].

The impact of water kefir-fermented soy milk (FSM) on key metabolic enzymes and physiological parameters in rats that were fed a high-fat fructose diet (HFFD) has also been explored. FSM produced by fermentation based on water kefir effectively inhibited pancreatic lipase and α-amylase activities, crucial for lipid and carbohydrate digestion, respectively, with the most significant inhibition observed after 16 hours of fermentation. In HFFD-fed rats, intestine and pancreas lipase and α-amylase activities are increased, leading to elevated plasma lipid levels, blood glucose, and weight gain. However, FSM supplementation reversed these effects by reducing enzyme activities, decreasing plasma total cholesterol and LDL-cholesterol, increasing HDL-cholesterol, and mitigating weight gain. Moreover, FSM protected against liver and kidney dysfunction induced by the HFFD. These findings highlight FSM's potential as a functional food to counteract obesity-related metabolic disturbances by modulating lipid and carbohydrate metabolism, improving lipid profiles, and safeguarding against organ toxicity associated with high-fat diets [274].

## 4. Conclusions, limitations and future perspectives

Nowadays, consumers have become increasingly concerned about incorporating healthy foods into their diet, including probiotics and traditional fermented beverages. In this direction, water kefir demand is increasing as an alternative to a fermented food based on a non-dairy matrix with potential health properties. The health promoting properties may depend on the microorganisms that are present in the beverage and/or the metabolites produced during fermentation. Within this study, the health promoting properties of the most representative microorganisms present in water kefir, as well as the health benefits attributed to the bioactive metabolites produced by each individual MO, were thoroughly reviewed, with emphasis given to the antioxidant, antithrombotic, and anti-inflammatory bio-functionalities of both MO and their metabolites. Moreover, an extensive presentation of the antioxidant and anti-inflammatory health benefits observed from the overall water kefir cultures and classic water kefir beverages obtained was also conducted. Finally, the use of water kefir for the production of several other functional products and applications with anti-inflammatory and antioxidant health promoting potential was also thoroughly discussed.

It should be noted that water kefir grains and the corresponding fermented beverage usually contain the same species, but these MO and their produced metabolites may differ in their relative abundance. However, as all the previously mentioned reports were obtained studying different beverages, each one obtained with different grains and different fermentation conditions that affect the microbial and chemical composition, it cannot be concluded that a specific water kefir may exert all these benefits, and more research is needed.

The difference in beverage microbiota is mainly related to the grain inoculum used for the fermentation as well as fermentation conditions. Furthermore, the non-dairy substrate (fruit juice, soy, etc.) added during water kefir fermentation may contribute to significant changes in the microbial diversity and the produced metabolites, leading to their overall bio-functionality. Thus, fermentation conditions affect metabolites that are produced, which is closely related to the biological effect. These strain-specific bioactive components will provide different potential health benefits depending on their nature and mode of action, which in many cases needs to be elucidated. Also, more research is needed to understand water kefir microbial interactions in a specific substrate and how these may affect the metabolites produced and the associated health benefits.

**Figure 1.**
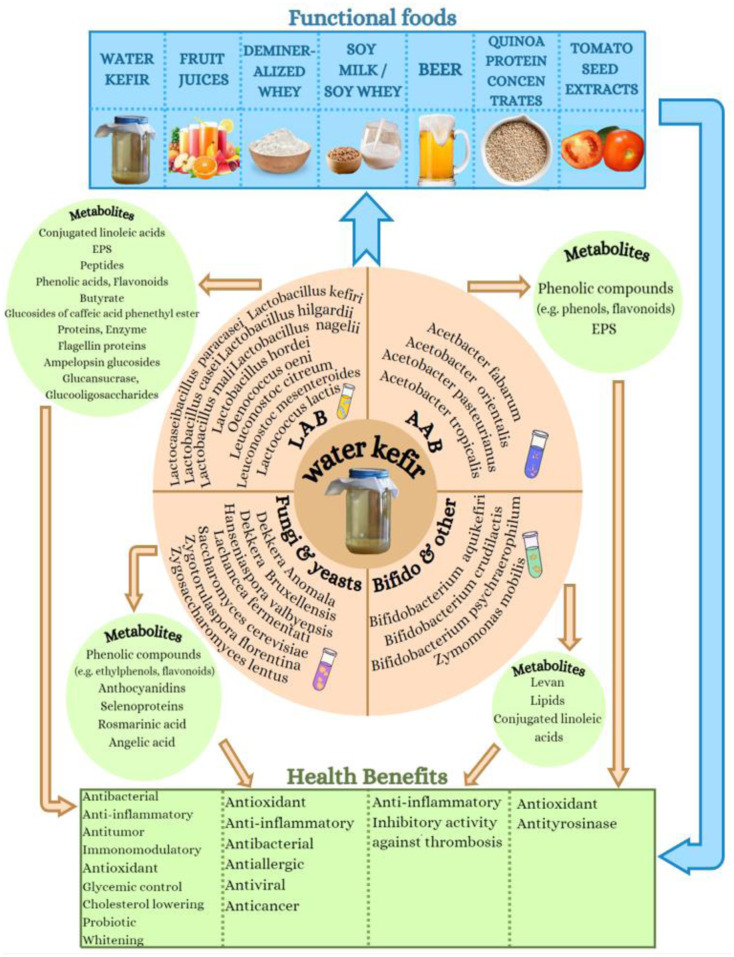
The microbiota and bioactives of water kefir and its fermented functional products with anti-inflammatory, antioxidant and several other associated health promoting properties.

**Table 6.** Antioxidant and anti-inflammatory health promoting effects of water kefir fermented products.

Fermented Product	Health promoting property	Ref
Tomato Seed Extract	Enhanced antioxidant activity	[266]
Soy Whey	Bioactive beverage with antioxidant activity	[267],[273]
Various Fruit Juices	High antioxidant capacity	[268]
Pomegranate Juice	Increased antioxidant activity and total phenolic content	[93]
Quinoa Protein Concentrates	High total phenolic content	[108]
Demineralized Whey	High total phenolic content	[109]
Red Beetroot Juice	Increased antioxidant capacity	[272]
Beer	Increased anti-inflammatory activity	[264]
Orange Water Kefir	Antioxidant activity and renal cell damage protection	[266]
Soy Milk	Inhibition of pancreatic lipase and α-amylase activities, increase in HDL-cholesterol and reduction in plasma total cholesterol and LDL-cholesterol, as well as protection against liver and kidney dysfunction	[274]

Water kefir grains are necessary not only to produce water kefir drinks, but also as a source of other interesting metabolites like phenolics, polar lipids, EPS, and glucans that could be used as new materials for functional product development and/or industrial applications due to their techno-functional properties and strong bio-functionalities with health-promoting effects against inflammation related-manifestations.

Taking into consideration that the microbial ecosystem and the metabolites present in the water kefir are deeply dependent on processing variables such as the origin of the grain and fermentation conditions, which will consequently affect the health benefits ascribed to the fermented product as well as grain growth, studies about potential properties of microorganisms isolated from the grain and the formulation of defined starters could be proposed as an innovative strategy that would allow the elaboration of products with a constant quality.

Finally, the elaboration of water kefir for fermentation of agri-food by-products as substrates in standardized fermentation conditions with specified water kefir grains can also contribute to the valorization of these bio-wastes, which may contribute to the development of eco-friendly, innovative bio-functional products with health-promoting and techno-functional properties for several foods, cosmetics, and pharmaceutical industrial applications in a circular economy design.

## Author contributions

Conceptualization, A.T.; writing—original draft preparation, all authors; writing—review and editing, AT; visualization, A.T.; supervision, A.T.; project administration, A.T. All authors have read and agreed to the published version of the manuscript.
